# Proteomic characterization of *van*A-containing *Enterococcu*s recovered from Seagulls at the Berlengas Natural Reserve, W Portugal

**DOI:** 10.1186/1477-5956-8-48

**Published:** 2010-09-21

**Authors:** Hajer Radhouani, Patrícia Poeta, Luís Pinto, Júlio Miranda, Céline Coelho, Carlos Carvalho, Jorge Rodrigues, María López, Carmen Torres, Rui Vitorino, Pedro Domingues, Gilberto Igrejas

**Affiliations:** 1 Institute for Biotechnology and Bioengineering, Center of Genomics and Biotechnology, University of Trás-os-Montes and Alto Douro, Vila Real, Portugal; 2 Department of Genetics and Biotechnology, University of Trás-os-Montes and Alto Douro; Vila Real, Portugal; 3 Center of Studies of Animal and Veterinary Sciences, Vila Real, Portugal; 4 Veterinary Science Department, University of Trás-os-Montes and Alto Douro, Vila Real, Portugal; 5 Biochemistry and Molecular Biology Area, University of La Rioja, Logroño, Spain; 6 Chemistry Department, University of Aveiro, Aveiro, Portugal

## Abstract

**Background:**

Enterococci have emerged as the third most common cause of nosocomial infections, requiring bactericidal antimicrobial therapy. Although vancomycin resistance is a major problem in clinics and has emerged in an important extend in farm animals, few studies have examined it in wild animals. To determine the prevalence of *van*A-containing *Enterococcus *strains among faecal samples of Seagulls (*Larus cachinnans*) of Berlengas Natural Reserve of Portugal, we developed a proteomic approach integrated with genomic data. The purpose was to detect the maximum number of proteins that vary in different enterococci species which are thought to be connected in some, as yet unknown, way to antibiotic resistance.

**Results:**

From the 57 seagull samples, 54 faecal samples showed the presence of *Enterococcus *isolates (94.7%). For the enterococci, *E. faecium *was the most prevalent species in seagulls (50%), followed by *E. faecalis *and *E. durans *(10.4%), and *E. hirae *(6.3%). *VanA-containing *enterococcal strains were detected in 10.5% of the 57 seagull faecal samples studied. Four of the *vanA*-containing enterococci were identified as *E. faecium *and two as *E. durans*. The *tet*(M) gene was found in all five tetracycline-resistant *vanA *strains. The *erm*(B) gene was demonstrated in all six erythromycin-resistant *vanA *strains. The *hyl *virulence gene was detected in all four *van*A-containing *E. faecium *isolates in this study, and two of them harboured the *pur*K1 allele. In addition these strains also showed ampicillin and ciprofoxacin resistance. The whole-cell proteomic profile of *van*A-containing *Enterococcus *strains was applied to evaluate the discriminatory power of this technique for their identification. The major differences among species-specific profiles were found in the positions corresponding to 97-45 kDa. Sixty individualized protein *spots *for each *vanA *isolate was identified and suitable for peptide mass fingerprinting measures by spectrometry measuring (MALDI/TOF MS) and their identification through bioinformatic databases query. The proteins were classified in different groups according to their biological function: protein biosynthesis, ATP synthesis, glycolysis, conjugation and antibiotic resistance. Taking into account the origin of these strains and its relation to infectious processes in humans and animals, it is important to explore the proteome of new strains which might serve as protein biomarkers for biological activity.

**Conclusions:**

The comprehensive description of proteins isolated from vancomycin-resistant *Enterococcus faecium *and *E. durans *may provide new targets for development of antimicrobial agents. This knowledge may help to identify new biomarkers of antibiotic resistance and virulence factors.

## Background

*Enterococcus *spp. are commensal bacteria of the intestinal microbiota of humans and animals but are now becoming recognized as important causes of nosocomial, and to a lesser extent, community acquired infections. Typical enterococcal infections occur in hospitalized patients with underlying conditions representing a wide spectrum of severity of illness and immune modulation [1,2]. The emergence of vancomycin-resistant enterococci (VRE) in Europe has been associated with the use of avoparcin as feed additive in food animals [3], until its ban in 1997 by the European Union. There are reports on the presence of VRE in farm animals in different countries [3-6], including in Portugal, but studies dealing with the occurrence of VRE in wild animals are limited [7,8]. For many years, vancomycin was considered as the last resort when all other classes of antibiotics failed. In the nineteen-eighties plasmid-mediated resistance against vancomycin among enterococci was first demonstrated and since then occurrences of infection caused by VRE have increased dramatically. This situation causes several challenges, including firstly the sole availability of expensive new antimicrobials for therapy of VRE infections since most strains are also resistance to multiple other economically acceptable drugs in developing countries, e.g., aminoglycosides or ampicillin, and secondly the possibility that the vancomycin resistance genes present in VRE could be transferred to other gram-positive microorganisms such as *Staphylococcus aureus *[9]. On the other hand virulence factors have been mainly detected in bacteria of the *E. faecalis *species, being *E. faecium *generally free of these determinants [10]. Studies reporting the presence of virulence factors in enterococci of food and animal origin are few [10,11], and the occurrence is not well documented in faecal enterococci from wild animals [9,12,13]. Birds are sentinel species whose plight serves as barometer of ecosystem health and alert system for detecting global environmental ills. Harmful effects seen in wildlife can be useful 'sentinel events' warning us of potential hazards for humans. This calls for integrated ecological and health hazard appraisals. Frequently, these wild birds are often opportunistic marine feeders along the shoreline or offshore, but also readily utilizing the food sources provided by humans, especially garbage. Migrating birds that fly and travel long distance seem to act as transporters, or as reservoirs, of resistant bacteria and may consequently have a significant epidemiological role in the dissemination of resistance, as well as being mirrors of the spectrum of pathogenic microorganisms present in humans. Of such migratory birds, particularly dominant in our study were the seagulls.

A natural heritage of great environmental value, the Berlengas archipelago, is situated about 10 km from the Portugal Peniche coast. It comprises the Berlengas Grande Island and adjacent reefs. It has been classified as a Natural Reserve since 1981. In an almost pure and wild state, the archipelago is a rich habitat for many animals and plant species. The dominant local fauna consists mainly of sea birds. Nowadays, the archipelago has no permanent human population and is only visited by scientists and, in the summer, by a small number of tourists. Visitors are required to respect the natural environment and the species that inhabit the area. Makeshift paths are marked with stones and park rangers watch out for visitors straying into the prohibited areas, disturbing the birds and the wildlife of this reserve. For these reasons, apparently, the seagulls are not directly under antibiotic selective pressure in the Berlengas.

Polyacrylamide gel electrophoresis (PAGE) of whole-cell polypeptides solubilised by treatment with sodium dodecyl sulfate (SDS) has been used to identify and type bacteria [14-16]. This technique allows the comparative study of large numbers of proteins encoded by a significant portion of the genome and, therefore, has a very high potential for measuring relationships among isolates [15,17,18]. Over the past decade numerous genomes of pathogenic bacteria were fully sequenced and annotated, while others are continuously being sequenced and published. More recently to understand the molecular mechanisms of bacteria resistance to glycopeptides, proteomic profiles of vancomycin-resistant *Enterococcus faecalis *V583 (reference strain) and V309 (clinical isolate) were analysed [19]. Vancomycin induced specifically and reversibly VanA, VanX, VanB, and VanXB. Some of these proteins have known vancomycin resistance functions or are related to virulent factors, stress, metabolism, translation, and conjunction, which would help *Enterococcus *survive under drug selection.

The genetic characterization of antimicrobial resistance genes as well as their location and diversity is important in identifying factors involved in resistance, understanding the diversity of multi drug resistant strains, identifying genetic linkages among markers, understanding potential transfer mechanisms, and developing efficient detection methods. The aim of this study was to analyse the prevalence of faecal carriage by *vanA*-containing *Enterococcus *strains in seagulls (*Larus cachinnans*) inhabiting the Berlengas archipelago, which are a group of very small islands of the Portuguese coast near to the city of Peniche. The islands are one of the first protected areas in the world. Additionally *esp *and *hyl *virulence factor genes were also investigated. Unlike genome studies, investigations at the proteomic level provide insights into protein abundance and/or post-translational modifications and it is also one of the best methods of investigating basic biological processes such as pathogenesis, physiology, and metabolic mechanisms. For these reasons the whole-cell protein (WCP) profiles of *vanA*-containing *Enterococcus *strains was followed by genotypic and proteome characterization of these bacteria. The goal was to demonstrate the usefulness of the WCP profiling approach as a technique for identifying, typing and studying the relationships between isolates.

## Results

### Phenotypic and genetic characterization of enterococci isolates to antibiotic resistance

Additional file 1 shows the different antimicrobial resistance genotypes detected in the enterococci isolates showing resistance to one or more antibiotic agents recovered from seagulls. It demonstrates that the majority of the enterococci strains carried the combination *tet*(M) + *tet*(L) and *erm*(B) genes. Different genomic profiles (17) were demonstrated in all of the enterococci isolates. The most prevalent genotype included the *tet*(M) + *tet*(L) + Tn*916 *+ Tn*5397 *+ *erm*(B) genes.

*vanA*-containing enterococcal strains were detected in six of the 57 seagull samples (10.5%). Four of the *vanA*-containing *Enterococcus *strains were identified as *E. durans*, and two as *E. faecium*. The characteristics of these strains and of the animals from which they were recovered are shown in additional file 2. All *vanA *strains showed high level vancomycin (MIC ≥128 mg/L), and teicoplanin resistance (MIC 64 mg/L); most of them showed resistance for tetracycline (n = 5), and all of them for erythromycin. The *tet*(M) gene was found in all five tetracycline-resistant *vanA *strains, in most of the cases associated with *tet*(L) gene (additional file 2); the *erm*(B) gene was demonstrated in all six erythromycin-resistant *vanA *strains. Enterococci with intrinsic vancomycin resistance (*van*C-1 or *vanC*2-3 gene) were not found. The *esp *gene was not detected among our isolates. The *hyl *virulence gene was detected in both *van*A-containing *E. faecium *isolates in this study (additional file 2), and they harboured the *pur*K1 allele, and in addition showed ampicillin and ciprofoxacin resistance. Additionally, the MLST typing of the two *van*A-positive *E. faecium *isolates demonstrated the ST5 type (included in CC17 clonal complex).

### One-dimensional electrophoresis

**Figure 1:**
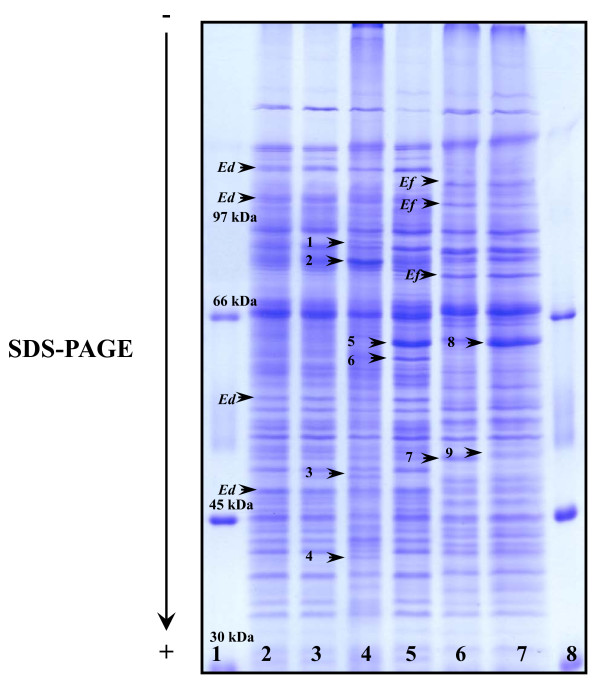
**SDS-PAGE of vancomycin-resistant enterococcal strains**. Lanes 1 and 8: Molecular mass markers (LMW Pharmacia kit); Lanes 2 to 5: *E. durans *(SG1, SG2, SG3 and SG56, respectively); Lanes 6 and 7: *E. faecium *(SG41 and SG50, respectively). Legend: Ef: *E. faecium*; Ed: *E. durans*. Numbers 1 to 8 represents bands which constitute the major observable differences between enterococci strains.

The enterococci isolates were displayed in the one-dimensional gel electrophoresis based on the genotypic profiles similarities. The SDS-PAGE of whole-cell extracts of the 6 *vanA-*containing enterococci strains are shown in Figure 1. Analysis of different strains by SDS-PAGE gave reproducible whole-cell proteins patterns which allowed differentiation between the species included in this study (*Ed *for *E. durans *and *Ef *for *E. faecium*). The major differences between these two species were identified in the 97-45 kDa region. The four *E. durans *strains studied presented two different genomic patterns (*tet*(M)-*tet*(L)-*erm*(B) and *tet*(M)-*tet*(L)-*erm*(B)-*hyl*), were traduced into three different protein profiles. This picture reveals the higher complexity of the proteome when compared with the static genome screened by PCR. Similar antibiotic resistance had similar protein profiles for *E. durans *(SG 1 and SG 2) (Figure 1, Lanes 2 and 3) but shows evident differences when compared strains SG3 and SG56, both identified as *E. durans *(Figure 1, Lanes 4 and 5). Although these two strains show the same genotypic pattern (*tet*(M)-*tet*(L)-*erm*(B)-*hyl*) presents very clear differences between protein bands (1-4 in contrast to 5-6). Differences revealed by SDS-PAGE for the two *E. faecium *strains characterised which have differences in tetracycline gene, SG 41 (*erm*(B)-*hyl*) and S-G 50 (*tet*(M)-*tet*(L)-*erm*(B)-*hyl*) were represented by three different bands (7-9).

### Two-dimensional Electrophoresis

**Figure 2:**
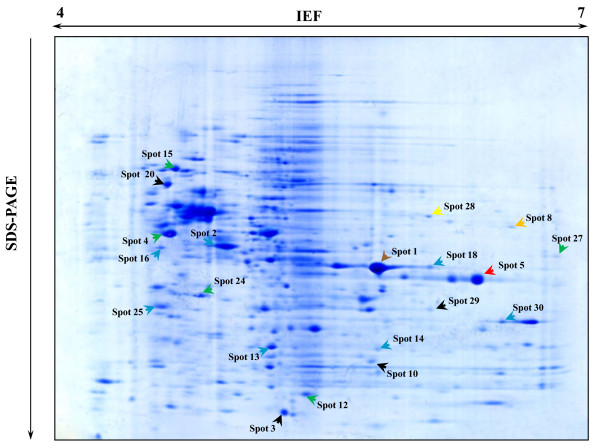
**2-DE gel image of SG 3 VRE with IPG strips pH4-7**. Legend: Green: Protein biosynthesis; Yellow: ATP synthesis; Blue: Glycolysis; Brown: Conjugation; Red: Antibiotic resistance; Black: Proteins of vanA E. durans SG 3 isolate with different biological processes from the proteins of *van*A *E. faecium *SG 41 isolate.

**Figure 3:**
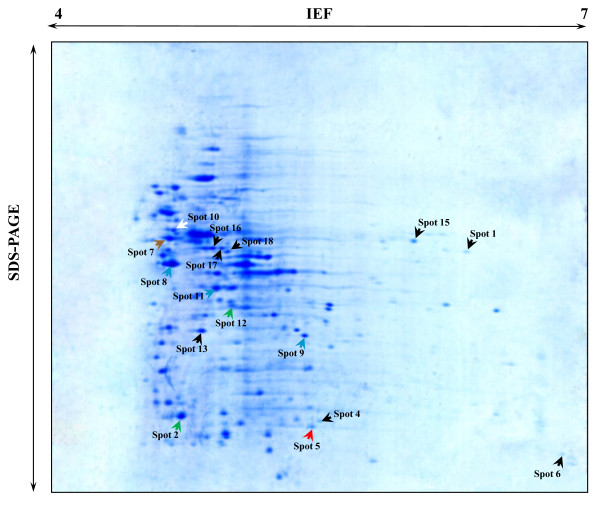
**2-DE gel image of SG 41 VRE with IPG strips pH4-7**. Legend: Green: Protein biosynthesis; Blue: Glycolysis; Brown: Conjugation; Red: Antibiotic resistance; Black: Proteins of *van*A *E. faecium *SG 41 isolate with different biological processes from the proteins of vanA *E. durans *SG 3 isolate.

A comparative analysis among the strains has been carried out. The protein expressions of the two vancomycin-containing enterococci (*vanA E. faecium *SG 41 and *vanA E. durans *SG 3) strains were visualized on 2-DE gels (Figures 2 and 3). The use of pH 4-7 IPG strips resulted in a well spread protein spots display which contributed to an accurate and safe excision and image identification of the spots. For each sample SG 3 and SG 41, a total of 60 relevant protein *spots *were collected for their analysis using MALDI-TOF mass spectrometry.

**Figure 4:**
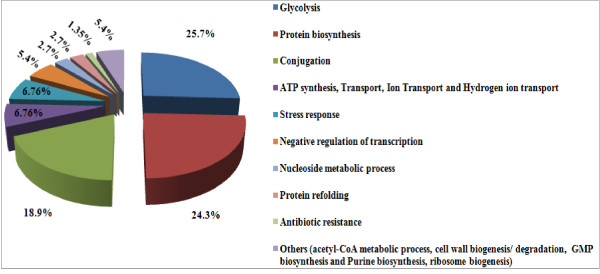
**Distribution of the biological processes related to the protein spots found in the 2-DE gel of the *vanA E. durans *SG 3 isolate**.

**Figure 5:**
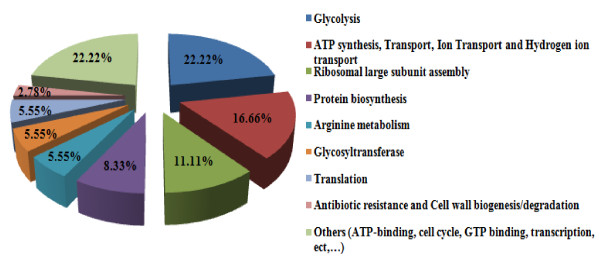
**Distribution of the biological processes related to the protein spots found in the 2-DE gel of the *vanA E. faecium *SG 41 isolate**.

**Figure 6:**
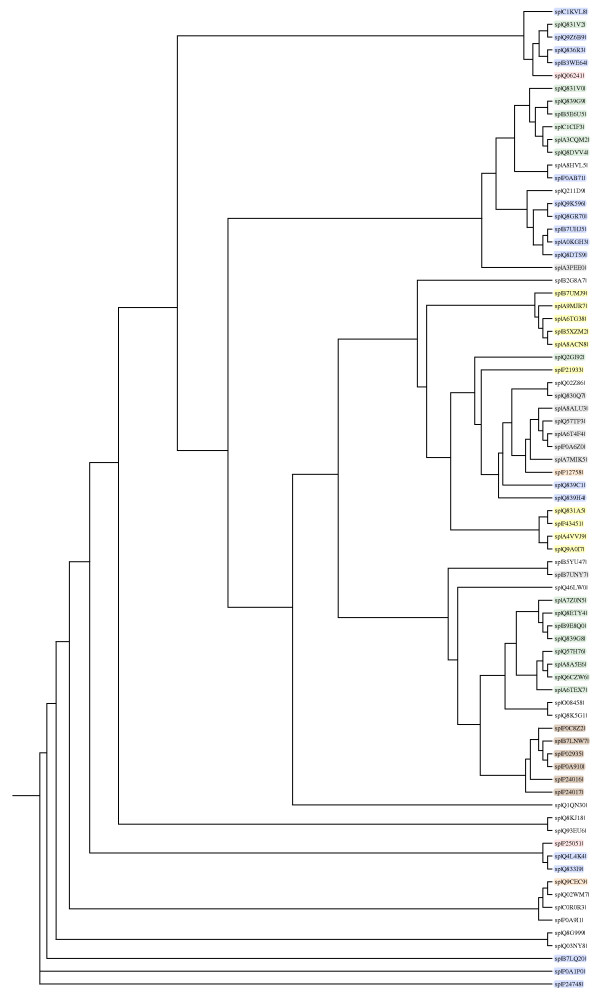
**Phylogenetic tree of FASTA protein sequences of all proteins identified**. The full alignment of these sequences were done with MUSCLE (v3.7) configured for highest accuracy. Legend: Green: Protein biosynthesis; Yellow: ATP synthesis; Blue: Glycolysis; Brown: Conjugation; Red: Antibiotic resistance; Black: Proteins of *vanA E. durans* SG 3 isolate with different biological processes from the proteins of *van*A strains.

The peptide mass peaks were compared with those in the NCBI database http://www.ncbi.nlm.nih.gov/, and the protein identification data including genebank ID, MW, PI value, mascot score, number of matched peptides and sequence coverage ratio (%) are listed in additional file 3 for and *vanA E. durans *SG 3 and additional file 4 for the *vanA E. faecium *SG 41 proteins. The identified proteins were showing diverse functional activities including glycolysis, conjugation, translation, protein biosynthesis, among others (Figures 4 and 5). Replicate sequences, truncated sequences, and sequences with partial alignments were removed from the BLAST results (not shown). From the collected sequences were selected to represent the initial tree. These sequences were aligned, and a phylogenetic tree was constructed by the minimum-evolution method to root the tree. The clustering of the initial phylogenetic tree indicated that all of the proteins included in the data set diverged from a common ancestor (Figure 6).

## Discussion

Unlike the genome, the proteome is dynamic: it varies according to the cell type and the functional state of the cell. In addition, the proteome shows characteristic perturbations in response to disease and external stimuli [20]. Nevertheless three different bands were observed (7 until 9) which can represent hypothetic correlation with differences in genomic data. The molecular weight of these bands is similar to those obtained from the 2-DE separations, which identify nine proteins associated with resistance to tetracycline [21]. The *esp *gene, that encodes an enterococcal surface protein associated with the ability to biofilm formation on abiotic surfaces [10], is more frequently found in clinical isolates [22], and this fact could explain the absence of detection among the commensal isolates analysed in this study. The wide dissemination of vancomycin-resistant *E. faecium *isolates of the epidemic-virulent clonal complex-17 (CC17), which harbour the *pur*K1 allele, and are very frequently associated with ampicillin and ciprofloxacin resistance, and also with the presence of *esp *and *hyl *virulence genes has been reported [23-26]. Two of our isolates present most of these characteristics (although do not harbour *esp *gene).

In our study of the identified proteins, it is important to point out the presence of vancomycin/teicoplanin A-type resistance protein *vanA *in *vanA-E. durans *SG 3 isolate. It has therefore been postulated that resistant cells produce peptidoglycan precursors that terminate in the depsipeptide D-alanine-2-D-hydroxy acid rather than the dipeptide D-alanine-D-alanine, thus preventing vancomycin binding [27]. Vancomycin-dependence results from a mutation that inactivates the D-Ala: D-Ala ligase gene (*ddl*) in the chromosome, so that the mutant strain no longer produces D-Ala: D-Ala-ending peptidoglycan precursors. Thus, cell wall synthesis in the mutant strain is dependent on the production of alternative peptidoglycan precursors. The D-Ala: D-Lac ligase activity of *vanA *and *vanB *can replace ddl activity by production of D-Ala: D-Lac-ending peptidoglycan precursors instead of the native D-Ala: D-Ala-ending precursors. As both resistances are inducible with vancomycin, the production of alternate precursors requires the presence of vancomycin and the mutant strain becomes vancomycin-dependent for growth [28]. It is interesting to find the ddl protein in *vanA E. durans *isolate from faecal sample of seagulls because since *E. faecalis *and *E. faecium *represent more than 95% of the clinical isolates collected, identification of enterococci based on the amplification of a fragment internal to the *ddl *gene encoding a D-Ala-D-Ala ligase included only these two species [29].

In *vanA E. faecium *SG 41 isolate it is notice to indicate the presence of D-alanyl-D-alanine dipeptidase that hydrolyzes D-Ala-D-Ala, thereby preventing vancomycin binding. The unstability of depsipeptide could also make VanY, a D-, D-carboxypeptidase in the *van *gene cluster which could participate to vancomycin resistance by removing D-Ala residue from C-terminus of peptidoglycan [30], functionally unnecessary for high-level vancomycin resistance in *E. faecium *isolate.

From a total of 60 protein spots identified in *vanA E. durans *SG 3 isolate, 5 proteins were found as related to stress response. Chaperone protein dnaK was detected and shows to be involved in the stress response mechanism for heat, a very important reaction for the survival of bacteria such as enterococci and that contributes for the antibiotic resistance capability [31]. The protein dnaK was detected in spot 20 as linked to two *E. coli *serotype 0157:H7 strains (accession number P0A6Z0 and A6T4F4), one *Citrobacter koseri *strain (A8ALU3), one *Enterobacter sakazakii *strain (A7MIK5) and one *Salmonella choleraesuis *strain (Q57TP3). In the spot 21 was found the protein 60 kDa chaperonin (groL) in the *vanA E. durans *SG 3 isolate related to *Enterococcus faecalis *(Q93EU6) and *Streptococcus constellatus *(Q8KJ18). The protein groL was also present in the spot 10 of the *vanA E. faecium *SG 41 isolate. This protein prevents misfolding and promotes the refolding and proper assembly of unfolded polypeptides generated under stress conditions [32,33].

The D-alanine-D-alanine ligase protein (A3PEE0) was also found in this isolate (SG 41) as being related with *Prochlorococcus marinus*, where it is involved in the cell wall biogenesis and peptidoglycan biosynthesis [34]. It is important to highlight the presence in the *vanA E. durans *SG 3 isolate of the wrbA flavoprotein (B7UNY7 and B5YU47) related to two enterhemorrhagic *E. coli *strains (*Escherichia coli *O127:H6 and *Escherichia coli *O157:H7, respectively). WrbA (tryptophan [W] repressor-binding protein) was discovered in *Escherichia coli*, where it was proposed to play a role in regulation of the tryptophan operon. This protein seems to improve the formation and/or stability of noncovalent complexes between the trp repressor protein and operator-bearing DNA [35,36]. This wrbA flavoprotein was also detected in *E. coli *C580 isolated from faecal sample of human by our investigation group and shows the partage of different sequences among different bacteria [37].

Our results clearly show that electrophoretic methods can provide valuable epidemiological information that may be used to isolate and characterize *Enterococcus *spp. The results are in accordance with previous results of Wang et al. [19] that demonstrated that many proteins involved in antibiotic resistance were differentially regulated by vanomycin which also triggered innate signal regulators, adhesion factors, and metabolic gene expression in *E. faecalis*. Therefore, these responses may enable *Enterococcus *spp. to adapt, survive, and remain pathogenic even under pressure of vancomycin treatment.

## Conclusions

This work, albeit preliminary in nature, reveals the complexity of expressed proteins in bacteria or different species and profiles of antibiotic resistance. SDS-PAGE patterns can be obtained easily and rapidly, are reproducible and do not require any sophisticated equipment and expensive reagents. Although protein profiles also represent phenotypic characteristics, they are considered to provide an excellent approximation of a microorganism's genome information. In addition, their extractability, sequence homology, and post translational modifications (PTMs) make proteomic analysis complex and informative. Proteomic methodologies contribute towards determining antimicrobial resistance mechanism(s) through the capacity to analysis global changes of bacteria. The totality of proteins identified in the present work are not necessarily related with antibiotics, hence the importance of 2DE. Epidemiological studies in different animals should be continued in the future to elucidate the evolution of *vanA *enterococcal and enterococci strains in different ecosystems. The complete sequencing and comparative proteome of some of these strains were isolated for the first time in this wild population of seagulls as well as the recognition of these proteins as markers in antibiotic resistance mechanisms. This biochemical and genomic foundation coupled to the parallel improvements of proteomic procedures enabled us to study *van*A-enterococci proteome. This now provides a sound basis for a comprehensive understanding of adaptability to environment and pathogenicity mechanisms. This work reports the impact of proteomics on our knowledge of *van*-A enterococci strains.

To our knowledge, this study is the first report which identifies candidate proteins related in antibiotic resistance and involved in the general stress response in *vanA*- containing *Enterococcus faecium *and *durans *species. Therefore, our results may reflect the expression of a few membrane proteins involved in antibiotic resistance. Correlation with web databases allowed the exact identification and characterization of the proteins present as well as their functions and relations within known biological processes occurring at the cellular level in enterococci. Proteomics and protein identification by 2-DE correlated with MALDI/TOF-TOF and bioinformatic databases are expected to become increasingly essential in elucidating the mechanisms of antibiotic resistance.

## Materials and methods

### Samples and bacteria

The presence of faecal VRE was investigated in 57 faecal samples recovered from seagulls of Berlengas islands. Faecal samples of seagulls were recovered in the soil along the entire Berlengas Island during September of 2007 and they were tested for the presence of *vanA*-containing *Enterococcus *isolates.

Faecal samples were diluted and sampled in Slanetz-Bartley agar plates, incubated 48 h at 35°C, and two different colonies were isolated but only a single isolate of each species was included. Colonies with typical enterococcal morphology were identified to the genus and species level by cultural characteristics, Gram's strain, catalase test, bile-aesculin reaction and by biochemical tests using the API ID20 Strep system (BioMérieux). Species identification was confirmed by PCR using primers and conditions for the different enterococcal species [6].

### Antimicrobial susceptibility testing

Antibiotic susceptibility was tested for 11 antibiotics of interest in animal and human medicine (μmug/disk): vancomycin (30), teicoplanin (30), ampicillin (10), streptomycin (300), gentamicin (120), kanamycin (120), chloramphenicol (30), tetracycline (30), erythromycin (15), quinupristin-dalfopristin (15), and ciprofloxacin, (5), by the disk diffusion method [38]. Antibiotic disks were obtained from Oxoid (Oxoid Ltd, Basingstoke, UK), with the exception of aminoglycoside disks that were prepared in the laboratory. Minimal inhibitory concentrations (MICs) of vancomycin (Eli Lilly, Indianapolis, IN, USA) and teicoplanin (Hoeschst Marion Roussell, Paris, France) were determined by the agar dilution method according to the CLSI (CLSI 2007). Serial two-fold dilutions were tested for antibiotic MIC determinations (from 0.25 μmug/ml to 64 μmug/ml). The breakpoints for resistance were the following ones: vancomycin or teicoplanin, ≥32 μmug/ml (a MIC of 8-16 μmug/ml of vancomycin or 16 μmug/ml of teicoplanin was considered as intermediate susceptibility). Only high-level resistance to aminoglycosides was considered in the susceptibility of our enterococci. *E. faecalis *strain ATCC29212 and *Staphylococcus aureus *strain ATCC29213 were used for quality control.

### Antimicrobial resistance genes

Vancomycin resistance genes (*van*A, *van*B, *van*C-1, *van*C-2/3 and *van*D) were tested by PCR in all vancomycin-resistant enterococcal strains, and positive amplicons were sequenced (Torres et al., 2003). Resistance genes for other antibiotics, including *tet*(M), *tet*(L), *erm*(A), *erm*(B), *erm*(C), were analysed by PCR [6]. The presence of *esp *and *hyl *virulence factor genes was tested by PCR in all isolates, using primers and conditions previously described [23], and the *purK *allele type was investigated by PCR and sequencing in all *E. faecium *isolates. Positive and negative controls were included in all analyses and the bacteria come from the collection of the University of Rioja (Spain). The specific gene harboured by the positive controls used in this study had previously been confirmed by sequencing, in all the cases.

### MLST typing

*E. faecium van*A isolates were characterized by Multilocus Sequence Typing (MLST). For this purpose, internal 400- to 600-bp fragments of seven housekeeping genes (*adk*, *atpA*, *ddl*, *gdh*, *gyd*, *purK *and *pstS*) were amplified and sequenced. The sequences obtained were analysed and compared with the database http://www.mlst.net. The combination of the seven obtained alleles for each isolate, give us a specific sequence type (ST) and clonal complex (CC) [39].

### Protein extraction

Frozen *van*A-containing *Enterococcus *cell stocks were streaked onto Luria-Bertani (LB) plates and grown at 37°C. Single colonies of *van*A-containing *Enterococcus *strains were conducted in 250 mL of M9 minimal medium supplemented with 4 gL-1 of glucose in covered 1 L Erlenmeyer flasks at 37°C. Cells were harvested from the exponential phase in all experiments. The cells were pelleted down at 10,000 rpm at 4°C for 3 min. The pellet should be visible after spinning and resuspended in an equal volume of pre-warmed phosphate-buffered saline (PBS) pH 7.4 [40]. After new centrifugation pellet was suspended in 0.2 ml of SDS sample solubilization buffer. The sample was sonicated with an ultrasonic homogenizer. The disrupted cells were centrifuged in an Eppendorf microfuge at maximum speed (14,000g) for 30 minutes at 4°C. For SDS-PAGE experiment the supernatant was collected and resuspended in an equal volume of buffer containing 0.5 M Tris HCl pH 8.0, glycerol, SDS and bromophenol blue.

### One-dimensional electrophoresis and coloration

One-dimensional electrophoresis was conducted on vertical gel with SDS-polyacrylamide gels (T = 12.52%, C = 0.97%) in a Hoefer™SE 600 Ruby^® ^(Amersham Biosciences) unit, following Laemmli [41] with some specific modifications [42]. Electrophoresis was carried out with a constant current of 30 mA per gel until the dye-front reached the bottom of the gels which were stained with Coomassie Brilliant Blue R250 during 24 hours and washed in water overnight. It was then fixated in trichloroacetic acid 6% for four hours and in glycerol 5% for two hours [43]. Reproducibility of the SDS-PAGE technique for enterococci characterization was confirmed by the analysis of triplicate protein extracts in which cells grown independently had similar banding patterns.

### Two-dimensional electrophoresis and proteomics

2-DE was performed according to the principles of O'Farrell [44] but with IPG (Immobiline™pH Gradient) technology [45]. Protein samples of *vanA E. durans *isolate [SG 3 VRE] were used in parallel with those *vanA E. durans *isolate [SG 41 VRE] proteins. For IEF, precast IPG strips with linear gradient of pH 4-7 were passively rehydrated overnight (12 to 16 hours) in a reswelling tray with rehydration buffer (8 M urea, 1% CHAPS, 0.4% DTT, 0.5% carrier ampholyte IPG buffer pH 3-10) at room temperature IPG strips were covered with DryStrip Cover Fluid (Plus One, Amersham Biosciences). Lyses buffer [9.5 M urea, 1% (w/v) DTT, 2% (w/v) CHAPS, 2% (v/v) carrier ampholytes (pH 3-10) and 10 mM Pefabloc^® ^proteinase inhibitor] was added to the two *vanA-containing *enterococci isolates (1:1). Samples containing a total of 100 μg of protein were loaded into 13 cm IPG strips (pH 4-7 NL, Amersham Biosciences, UK) [43]. The sample solution was then applied in the previously rehydrated IPG strips pH4-7 by cup loading and then proteins were focused sequentially at 500 V for 1 h, 1000 V for 1 h, 8000 V for 2 h 30 and finally 8000 V incremented to 12505 V/h on an Ettan™IPGPhor II™(Amersham Biosciences, Uppsala, Sweden). Seven IEF replicate runs were performed according to Görg [45] and the GE Healthcare protocol for IPG strips pH 4-7 of 13 cm, in order to obtain the optimized running conditions, resulting in a final 5 h 25 hour run. Focused IPG strips were then stored at -80°C in plastic bags. Before running the second dimension, strips were equilibrated twice 15 minutes in equilibration buffer (6 M urea, 30% (w/v) glycerol, 2% (w/v) SDS in 0.05 M Tris-HCl buffer (pH 8.8)). In the first equilibration it was added 1% DTT to the original equilibration buffer and to the second 4% iodoacetamide, and also bromophenol blue was added to both solutions. The equilibrated IPG strips were gently rinsed with SDS electrophoresis buffer, blotted to remove excessive buffer, and then applied onto a 12.52% polyacrylamide gels in a Hoefer™SE 600 Ruby^® ^(Amersham Biosciences) unit. Some modifications were introduced in the SDS-PAGE technique previously reported by Laemmli [41], that allowed its resolution to be increased, with proper insertion of the IPG strips in the stacking gel [41,42]. After SDS-PAGE, the 2-DE gels were fixated in 40% methanol/10% acetic acid for one hour and afterwards stained overnight in Coomassie Brilliant Blue G-250 [40]. Coomassie-stained gels were scanned on a flatbed scanner (Umax PowerLook 1100; Fremont, CA, USA), and the resulting digitized images were analyzed using Image Master 5.0 software (Amersham Biosciences; GE Healthcare).

### Protein identification by MALDI-TOF/TOF

Spots of expression in all gels were manually excised from the gels and analyzed using Matrix-Assisted Laser Desorption/Ionization-Time of Flight Mass Spectrometry (MALDI-TOF). The gel pieces were washed three times with 25 mM ammonium bicabornate/50% ACN, one time with ACN and dried in a SpeedVac (Thermo Savant). 25 mL of 10 mg/mL sequence grade modified porcine trypsin (Promega) in 25 mM ammonium bicabornate was added to the dried gel pieces and the samples were incubated overnight at 37°C. Extraction of tryptic peptides was performed by addition of 10% of formic acid (FA)/50% ACN three times being lyophilised in a SpeedVac (Thermo Savant). Tryptic peptides were ressuspended in 10 mL of a 50% acetonitrile/0.1% formic acid solution. The samples were mixed (1:1) with a matrix consisting of a saturated solution of a-cyano-4-hydroxycinnamic acid prepared in 50% acetonitrile/0.1% formic acid. Aliquots of samples (0.5 μL) were spotted onto the MALDI sample target plate.

Peptide mass spectra were obtained on a MALDI-TOF/TOF mass spectrometer (4800 Proteomics Analyzer, Applied Biosystems, Europe) in the positive ion reflector mode. Spectra were obtained in the mass range between 800 and 4500 Da with ca. 1500 laser shots. For each sample spot, a data dependent acquisition method was created to select the six most intense peaks, excluding those from the matrix, trypsin autolysis, or acrylamide peaks, for subsequent MS/MS data acquisition. Mass spectra were internally calibrated with autodigest peaks of trypsin (MH+: 842.5, 2211.42 Da) allowing a mass accuracy of better than 25 ppm.

### Database search

Spectra were processed and analyzed by the Global Protein Server Workstation (Applied Biosystems), which uses internal MASCOT software (v 2.1.04, Matrix Science, London, UK) on searching the peptide mass fingerprints and MS/MS data. Swiss-Prot nonredundant protein sequence database was used for all searches under *Enterococcus*. Database search parameters as follows: carbamidomethylation and propionamide of cysteine (+71Da) as a variable modification as well as oxidation of methionine (+16Da), and the allowance for up to two missed tryptic cleavages. The peptide mass tolerance was 25 ppm and fragment ion mass tolerance was 0.3 Da. Protein identifications were considered as reliable when the MASCOT score was > 70 (MASCOT score was calculated as − 10 × log P, where P is the probability that the observed match is a random event.). This is the lowest score indicated by the program as significant (P < 0.05) and indicated by the probability of incorrect protein identification.

### Sequence alignments and construction of the phylogenetic tree

The analysis was performed on the Phylogeny.fr platform and comprised the following different steps. Sequences were aligned with MUSCLE (v3.7) configured for highest accuracy (MUSCLE with default settings). The phylogenetic tree was reconstructed using the maximum likelihood method implemented in the PhyML program (v3.0 aLRT). The JTT substitution model was selected assuming an estimated proportion of invariant sites (of 0.000) and 4 gamma-distributed rate categories to account for rate heterogeneity across sites. The gamma shape parameter was estimated directly from the data (gamma = 12.476). Reliability for internal branch was assessed using the aLRT test (SH-Like). Graphical representation of the phylogenetic tree (phenogram) was performed with Drawgram from the PHYLIP package (v3.66).

## Abbreviations

2-DE: two-dimensional polyacrylamide gel electrophoresis; PCR: Polymerase Chain Reaction; MALDI-TOF MS: matrix-assisted laserdesorption ionization time-of-flight mass spectrometry, spp.: subspecies; MIC: minimal inhibitory concentration.

## Competing interests

The authors declare that they have no competing interests.

## Authors' contributions

HR carried out sample preparation, SDS-PAGE and 2DE analysis. PP collected faecal samples and isolated VRE. LP helped in SDS-PAGE and 2DE. CC and JM carried out bioinformatic analyses. JR, ML, and CT helped the research. CC and CT also reviewed article. RV and PD carried MS/MS analyses. GI conceived, designed, implemented and coordinated the study. All authors read and approved the final manuscript.

## Supplementary Material

Additional file 1 **Antibiotic resistance genes in enterococci recovered from seagulls**. Different antimicrobial resistance genotypes detected in the enterococci isolates showing resistance to one or more antibiotic agents recovered from seagulls. Click here for file

Additional file 2 **Characteristics of vancomycin-resistant enterococcal strains recovered from seagulls in Berlengas**. *Enterococcus *strain, MIC, vancomycin resistant genes detected, resistant phenotype for other antibiotics, and resistance and virulence genes detected by PCR. Click here for file

Additional file 3 **Identification of proteins from *van*A *E. durans *SG 3 isolate using 2-DE gels and MALDI-TOF sequencing results**. Spot identification, protein description, species which was already isolated, protein name, accession number, protein MW and PI, peptide count, protein score, information about, and references (Additional file legend). Click here for file

Additional file 4 **Identification of proteins from *van*A *E. faecium *SG 41 isolate using2-DE gels and MALDI-TOF sequencing results**. Spot identification, protein description, species which was already isolated, protein name, accession number, protein MW and PI, peptide count, protein score, information about, and references (Additional file legend). Click here for file
