# Supplementary material for: Proteomic characterization of vanA-containing Enterococcus recovered from Seagulls at the Berlengas Natural Reserve, W Portugal
Source: Proteome Sci. 2010 Sep 21;8:48. doi: 10.1186/1477-5956-8-48 (PMC2954869; doi:10.1186/1477-5956-8-48)
Supplement: Additional file 1 — Antibiotic resistance genes in enterococci recovered from seagulls. Different antimicrobial resistance genotypes detected in the enterococci isolates showing resistance to one or more antibiotic agents recovered from seagulls. [file 1477-5956-8-48-S1.DOC]

**Additional file 1. Antibiotic resistance genes in enterococci recovered from seagulls.**

| Enterococal species | Resistance to antibiotics | Number of isolates resistant to this antimicrobial | Resistance genes detected | Number of isolates |
| --- | --- | --- | --- | --- |
| *E. faecium* | Tetracycline | 22 | *tet*(L) | 6 |
|  |  |  | *tet*(M)+*tet*(L) | 15 |
|  |  |  | Tn*916* | 3 |
|  |  |  | Tn*5397* | 3 |
|  |  |  | Tn*916*+ Tn*5397* | 7 |
|  | Erythromycin | 22 | *erm*(B) | 18 |
|  | Quinupristin-dalfopristin | 6 | *vat*(D) | 1 |
|  |  |  | *vat*(E) | 2 |
|  | Kanamycin | 7 | *aph*(3’)-IIIa | 7 |
|  | Streptomycin | 4 | *ant*(6)-Ia | 4 |
|  | Chloramphenicol | 3 | *cat*A | 0 |
| *E. faecalis* | Tetracycline | 4 | *tet*(L) | 1 |
|  |  |  | *tet*(M)+*tet*(L) | 3 |
|  |  |  | Tn*916*+ Tn*5397* | 1 |
|  | Erythromycin | 4 | *erm*(B) | 3 |
|  | Chloramphenicol | 2 | *cat*A | 2 |
|  |  |  |  |  |
| *E. hirae* | Tetracycline | 3 | *tet*(L) | 2 |
|  |  |  | *tet*(M)+*tet*(L) | 1 |
|  |  |  | Tn*916*/Tn*154* | 1 |
|  |  |  | Tn*5397* | 1 |
|  |  |  | Tn*916*+ Tn*5397* | 1 |
|  | Erythromycin | 3 | *erm*(B) | 3 |
|  | Quinupristin-dalfopristin | 1 | *vat*(D)/ *vat*(E) | 0 |
|  |  |  |  |  |
| *E. durans* | Tetracycline | 1 | *tet*(M)+*tet*(L) | 1 |
|  |  |  | Tn*916*+ Tn*5397* | 1 |
|  | Erythromycin | 1 | *erm*(B) | 1 |
|  |  |  |  |  |
| *Enterococcus* spp. | Tetracycline | 10 | *tet*(L) | 1 |
|  | Erythromycin | 10 | *erm*(B) | 1 |
|  | Quinupristin-dalfopristin | 5 | *vat*(D)*/ vat*(E) | 0 |
|  | Kanamycin | 2 | *aph*(3’)-IIIa | 2 |
|  | Streptomycin | 3 | *ant*(6)-Ia | 0 |
|  | Chloramphenicol | 1 | *cat*A | 0 |
