# Supplementary material for: Proteomic characterization of vanA-containing Enterococcus recovered from Seagulls at the Berlengas Natural Reserve, W Portugal
Source: Proteome Sci. 2010 Sep 21;8:48. doi: 10.1186/1477-5956-8-48 (PMC2954869; doi:10.1186/1477-5956-8-48)
Supplement: Additional file 2 — Characteristics of vancomycin-resistant enterococcal strains recovered from seagulls in Berlengas. Enterococcus strain, MIC, vancomycin resistant genes detected, resistant phenotype for other antibiotics, and resistance and virulence genes detected by PCR. [file 1477-5956-8-48-S2.DOC]

**Additional file 2. Characteristics of vancomycin-resistant enterococcal strains recovered from seagulls in Berlengas.**

| *Enterococcus* strain | MIC (in mg/L) to: | | Vancomycin resistant genes detected | *purK* allele | Resistant phenotype for other antibiotics^a^ | Resistance and virulence genes detected by PCR |
| --- | --- | --- | --- | --- | --- | --- |
|  | Vancomycin | Teicoplanin |  |  |  |  |
| *E. durans* SG1 | >128 | 64 | *vanA* | ND^b^ | TET - ERY | *tet*(M)*, tet*(L)*, erm*(B) |
| *E. durans* SG2 | >128 | 64 | *vanA* | ND | TET - ERY | *tet*(M)*, tet*(L), *erm*(B) |
| *E. durans* SG3 | >128 | 64 | *vanA* | 3 | TET - ERY-AMP | *tet*(M)*, tet*(L)*, erm*(B), *hyl* |
| *E. faecium* SG41 | >128 | 64 | *vanA* | 1 | ERY - CIP - AMP | *erm*(B), *hyl* |
| *E. faecium* SG50 | >128 | 64 | *vanA* | 1 | TET - ERY - CIP - AMP | *tet*(M)*, tet*(L)*, erm*(B), *hyl* |
| *E. durans* SG56 | 128 | 64 | *vanA* | ND | TET - ERY -AMP | *tet*(M)*, tet*(L)*, erm*(B), *hyl* |

^a^ AMP, ampicillin; CIP, ciprofloxacin; ERY, erythromycin; TET, tetracycline.

^b^ND: no determined
