# Supplementary material for: Proteomic characterization of vanA-containing Enterococcus recovered from Seagulls at the Berlengas Natural Reserve, W Portugal
Source: Proteome Sci. 2010 Sep 21;8:48. doi: 10.1186/1477-5956-8-48 (PMC2954869; doi:10.1186/1477-5956-8-48)
Supplement: Additional file 3 — Identification of proteins from vanA E. durans SG 3 isolate using 2-DE gels and MALDI-TOF sequencing results. Spot identification, protein description, species which was already isolated, protein name, accession number, protein MW and PI, peptide count, protein score, information about, and references (Additional file legend). [file 1477-5956-8-48-S3.DOC]

**Additional file 3:** Protein spots identification of 2-DE gels and MALDI-TOF sequencing results from *vanA E. durans* SG 3 isolate.

| ***Spot*** | ***Protein Description*** | ***Species*** | ***Protein Name*** | ***Accession Number*** | ***Protein MW*** | ***Protein PI*** | ***Peptide Count*** | ***Protein Score*** | ***Protein Score C.I. %*** | ***Information*** | ***References*** |
| --- | --- | --- | --- | --- | --- | --- | --- | --- | --- | --- | --- |
| 1 | **Outer membrane protein A** | ***Klebsiella pneumoniae*** | **ompA** | P24017 | 37038,3515625 | 5,73 | 10 | 89 | 100 | Involved in the action of colicins K and L and for the stabilization of mating aggregates in conjugation. Acted as a receptor for a number of T-even like phages. Also acts as a porin with low permeability that permits slow penetration of small solutes | 1, 2 |
| 2 | **Phosphoglycerate kinase** | ***Enterococcus faecalis*** | **pgk** | Q833I9 | 42371,01172 | 4,9 | 4 | 112 | 100 | Involved in the carbohydrate degradation; glycolysis; pyruvate from D-glyceraldehyde 3-phosphate: step 2/5. | 3 |
| 3 | **50S ribosomal protein L10** | ***Enterococcus faecalis*** | **rplJ** | Q830Q7 | 17611,53906 | 5,39 | 4 | 70 | 97 | Belongs to the ribosomal protein L10P family | 3 |
| 4 | **Elongation factor Tu** | ***Enterococcus faecalis*** | **tuf** | Q839G8 | 43360,69141 | 4,73 | 8 | 162 | 100 | During protein biosynthesis, this protein promotes the GTP-dependent binding of aminoacyl-tRNA to the A-site of ribosomes | 3 |
| 4 | **Elongation factor Tu** | ***Oceanobacillus iheyensis*** | **tuf** | Q8ETY4 | 43200,73047 | 4,81 | 5 | 140 | 100 |  | 4 |
| 4 | **Elongation factor Tu** | ***Bacillus amyloliquefaciens*** | **tuf** | A7Z0N5 | 43385,76172 | 4,84 | 4 | 134 | 100 |  | 5 |
| 4 | **Elongation factor Tu** | ***Macrococcus caseolyticus*** | **tuf** | B9E8Q0 | 43358,82031 | 4,78 | 8 | 127 | 100 |  | 6 |
| 5 | **Vancomycin/teicoplanin A-type resistance protein *vanA*** | ***Enterococcus faecium*** | ***vanA*** | P25051 | 37419,10938 | 5,79 | 15 | 368 | 100 | Involved in a high-level resistance to glycopeptide antibiotics. D-Ala--D-Ala ligase of altered specificity which catalyzes ester bond formation between D-Ala and various D-hydroxy acids; generates a peptidoglycan which does not terminate in D-alanine but in D-lactate, thus preventing vancomycin or teicoplanin binding | 7 |
| 6 | **Inosine-5'-monophosphate dehydrogenase** | *Streptococcus pyogenes* serotypeM3 | guaB | Q8K5G1 | 52773,26172 | 5,72 | 6 | 83 | 100 | Required for the purine metabolism; XMP biosynthesis via de novo pathway; XMP from IMP: step 1/1. | 8, 9 |
| 7 | **Outer membrane protein A** | ***Escherichia coli* (strain K12)** | **ompA** | P0A910 | 37177,66016 | 5,99 | 11 | 235 | 100 | Involved in the action of colicins K and L and for the stabilization of mating aggregates in conjugation. Serves as a receptor for a number of T-even like phages. Also acts as a porin with low permeability that allows slow penetration of small solutes. | 10, 11 |
| 7 | **Outer membrane protein A** | ***Escherichia fergusonii*** | **ompA** | B7LNW7 | 37678,89063 | 5,46 | 10 | 194 | 100 |  | 2 |
| 7 | **Outer membrane protein A** | ***Shigella dysenteriae*** | **ompA** | P02935 | 37717,89844 | 5,57 | 9 | 129 | 100 |  | 12 |
| 8 | **Uracil phosphoribosyltransferase** | ***Lactococcus lactis* subsp. *lactis*** | **upp** | Q9CEC9 | 23215,59961 | 6,54 | 8 | 155 | 100 | Involved in the pyrimidine metabolism; UMP biosynthesis via salvage pathway; UMP from uracil: step 1/1. | 13 |
| 9 | **Flavoprotein wrbA** | ***Escherichia coli* O127:H6** | **wrbA** | B7UNY7 | 20832,41992 | 5,59 | 11 | 122 | 100 | Seems to improve the formation and/or stability of noncovalent complexes between the trp repressor protein and operator-bearing DNA | 14 |
| 9 | **Flavoprotein wrbA** | ***Escherichia coli* O157:H7** | wrbA | B5YU47 | 20821,41992 | 5,91 | 9 | 90 | 100 |  | 15 |
| 10 | **Flavoprotein wrbA** | ***Escherichia coli* O127:H6** | **wrbA** | B7UNY7 | 20832,41992 | 5,59 | 10 | 122 | 100 |  | 14 |
| 10 | **Flavoprotein wrbA** | ***Escherichia coli* O127:H6** | **wrbA** | B7UNY7 | 20832,41992 | 5,59 | 9 | 88 | 100 |  |  |
| 11 | **Uridine phosphorylase** | ***Escherichia coli* (strain K12)** | **udp** | P12758 | 27141,83984 | 5,81 | 10 | 120 | 100 | Catalyzes the reversible phosphorylytic cleavage of uridine and deoxyuridine to uracil and ribose- or deoxyribose-1-phosphate. The produced molecules are then utilized as carbon and energy sources or in the rescue of pyrimidine bases for nucleotide synthesis. | 16, 17 |
| 12 | **Ribosome-recycling factor** | ***Enterococcus faecalis*** | **frr** | Q831V2 | 20790,96094 | 5,21 | 7 | 79 | 100 | Responsible for the release of ribosomes from messenger RNA at the termination of protein biosynthesis. Could augment the efficiency of translation by recycling ribosomes from one round of translation to another | 3 |
| 13 | **2,3-bisphosphoglycerate-dependent phosphoglycerate mutase** | ***Enterococcus faecalis*** | **gpmA** | Q839H4 | 25991,2207 | 5,09 | 8 | 176 | 100 | Involved in the carbohydrate degradation; glycolysis; pyruvate from D-glyceraldehyde 3-phosphate: step 3/5. | 3 |
| 14 | **Triosephosphate isomerase** | ***Enterobacter cloacae*** | **tpiA** | Q9Z6B9 | 26896,89063 | 5,77 | 6 | 100 | 100 | Participate in the carbohydrate degradation; glycolysis; D-glyceraldehyde 3-phosphate from glycerone phosphate: step 1/1. | 18 |
| 15 | **Elongation factor G** | ***Enterococcus faecalis*** | **fusA** | Q839G9 | 76630,47656 | 4,8 | 8 | 121 | 100 | This protein promotes the GTP-dependent translocation of the nascent protein chain from the A-site to the P-site of the ribosome. | 3 |
| 15 | **Elongation factor G** | ***Streptococcus mutans*** | **fusA** | Q8DVV4 | 76615,8125 | 4,81 | 8 | 112 | 100 |  | 19 |
| 15 | **Elongation factor G** | ***Streptococcus pneumoniae* serotype 19F** | **fusA** | B5E6U5 | 76782,77344 | 4,86 | 7 | 107 | 100 |  | 20 |
| 15 | **Elongation factor G** | ***Streptococcus sanguinis*** | **fusA** | A3CQM2 | 76730,7969 | 4,87 | 7 | 106 | 100 |  | 21 |
| 15 | **Elongation factor G** | ***Streptococcus pneumoniae*** | **fusA** | **C1CIF3** | 76754,7422 | 4,86 | 6 | 103 | 100 |  | 22 |
| 16 | Enolase | [*Enterococcus hirae*](http://www.uniprot.org/taxonomy/1354) | eno | Q8GR70 | 46382,4297 | 4,58 | 12 | 275 | 100 | Catalyzes the reversible conversion of 2-phosphoglycerate into phosphoenolpyruvate. It is essential for the degradation of carbohydrates via glycolysis | 23 |
| 16 | Enolase | [*Enterococcus faecalis*](http://www.uniprot.org/taxonomy/1351) | eno | Q9K596 | 46482,4805 | 4,56 | 8 | 215 | 100 | Catalyzes the reversible conversion of 2-phosphoglycerate into phosphoenolpyruvate. It is essential for the degradation of carbohydrates via glycolysis | 3 |
| 16 | Enolase | [*Streptococcus mutans*](http://www.uniprot.org/taxonomy/1309) | eno | Q8DTS9 | 46828,6094 | 4,67 | 6 | 196 | 100 |  | 19 |
| 17 | 6-phosphofructokinase | [*Enterococcus faecalis*](http://www.uniprot.org/taxonomy/1351) | pfkA | Q836R3 | 34390,6992 | 5,55 | 13 | 235 | 100 | [It involved in the carbohydrate degradation; glycolysis; D-glyceraldehyde 3-phosphate and glycerone phosphate from D-glucose: step 3/4.](http://www.grenoble.prabi.fr/obiwarehouse/unipathway?upid=UPA00109&entryac=Q836R3) | 3 |
| 17 | 6-phosphofructokinase | [*Listeria monocytogenes* serotype 4b](http://www.uniprot.org/taxonomy/634178) | pfkA | C1KVL8 | 34398,9492 | 5,46 | 6 | 80 | 100 |  | 24 |
| 18 | Fructose-bisphosphate aldolase class 2 | [*Escherichia coli*](http://www.uniprot.org/taxonomy/83333) | fbaA | P0AB71 | 39122,6094 | 5,52 | 5 | 69 | 96 | Catalyzes the aldol condensation of dihydroxyacetone phosphate (DHAP or glycerone-phosphate) with glyceraldehyde 3-phosphate (G3P) to form fructose 1,6-bisphosphate (FBP) in gluconeogenesis and the reverse reaction in glycolysis. | 25, 26 |
| 19 | Outer membrane protein A | *Escherichia coli* | ompA | P0A910 | 37177,6602 | 5,99 | 9 | 176 | 100 | Required for the action of colicins K and L and for the stabilization of mating aggregates in conjugation. Serves as a receptor for a number of T-even like phages. Also acts as a porin with low permeability that allows slow penetration of small solutes. | 26 |
| 19 | Outer membrane protein A | [*Escherichia fergusonii*](http://www.uniprot.org/taxonomy/564) | ompA | P0C8Z2 | 26128,2695 | 5,14 | 7 | 168 | 100 |  | 2 |
| 19 | Outer membrane protein A | [*Escherichia fergusonii*](http://www.uniprot.org/taxonomy/585054) | ompA | B7LNW7 | 37678,8906 | 5,46 | 8 | 167 | 100 |  | 2 |
| 19 | Outer membrane protein A | [*Shigella dysenteriae*](http://www.uniprot.org/taxonomy/622) | ompA | P02935 | 37717,8984 | 5,57 | 7 | 109 | 100 |  | 12 |
| 20 | Chaperone protein dnaK | [*Escherichia coli* O157:H7](http://www.uniprot.org/taxonomy/83334) | dnaK | P0A6Z0 | 69072,4766 | 4,83 | 20 | 167 | 100 | Acts as a chaperone | 27 |
| 20 | Chaperone protein dnaK | *Escherichia coli* O157:H7 | dnaK | A6T4F4 | 69066,4375 | 4,83 | 18 | 149 | 100 |  | 27 |
| 20 | Chaperone protein dnaK | [*Citrobacter koseri*](http://www.uniprot.org/taxonomy/290338) | dnaK | A8ALU3 | 69067,3281 | 4,81 | 18 | 149 | 100 |  | 28 |
| 20 | Chaperone protein dnaK | [*Enterobacter sakazakii*](http://www.uniprot.org/taxonomy/290339) | dnaK | A7MIK5 | 69174,4922 | 4,84 | 18 | 148 | 100 |  | 29 |
| 20 | Chaperone protein dnaK | [*Salmonella choleraesuis*](http://www.uniprot.org/taxonomy/28901) | dnaK | Q57TP3 | 69229,5313 | 4,84 | 17 | 139 | 100 |  | 30 |
| 21 | 60 kDa chaperonin | [*Enterococcus faecalis*](http://www.uniprot.org/taxonomy/1351) | groL | Q93EU6 | 57074,9297 | 4,64 | 15 | 170 | 100 | Prevents misfolding and promotes the refolding and proper assembly of unfolded polypeptides generated under stress conditions | 3 |
| 21 | 60 kDa chaperonin | [*Streptococcus constellatus*](http://www.uniprot.org/taxonomy/76860) | groL | Q8KJ18 | 56851,7109 | 4,63 | 13 | 134 | 100 |  | 31 |
| 22 | Elongation factor Tu 1 | [*Escherichia coli* O9:H4](http://www.uniprot.org/taxonomy/331112) | tuf1 | A8A5E6 | 43256,3086 | 5,3 | 9 | 86 | 100 | This protein promotes the GTP-dependent binding of aminoacyl-tRNA to the A-site of ribosomes during protein biosynthesis | 32 |
| 22 | Elongation factor Tu 1 | [*Salmonella choleraesuis*](http://www.uniprot.org/taxonomy/28901) | tuf1 | Q57H76 | 43256,3086 | 5,3 | 9 | 86 | 100 |  | 30 |
| 22 | Elongation factor Tu | [*Klebsiella pneumoniae* subsp. *pneumoniae*](http://www.uniprot.org/taxonomy/272620) | tufA | A6TEX7 | 43219,2617 | 5,29 | 8 | 78 | 100 |  | 33 |
| 23 | 6-phosphofructokinase | [*Enterococcus faecalis*](http://www.uniprot.org/taxonomy/1351) | pfkA | Q836R3 | 34390,6992 | 5,55 | 11 | 109 | 100 | [It involved in the carbohydrate degradation; glycolysis; D-glyceraldehyde 3-phosphate and glycerone phosphate from D-glucose: step 3/4.](http://www.grenoble.prabi.fr/obiwarehouse/unipathway?upid=UPA00109&entryac=Q836R3) | 3 |
| 24 | Elongation factor Ts | [*Enterococcus faecalis*](http://www.uniprot.org/taxonomy/1351) | tsf | Q831V0 | 32113,3301 | 4,87 | 9 | 87 | 100 | Associates with the EF-Tu.GDP complex and induces the exchange of GDP to GTP. It remains bound to the aminoacyl-tRNA.EF-Tu.GTP complex up to the GTP hydrolysis stage on the ribosome | 3 |
| 17 | 6-phosphofructokinase | [*Lactobacillus casei*](http://www.uniprot.org/taxonomy/543734) | pfkA | B3WE64 | 34208,5508 | 5,74 | 6 | 79 | 100 | [It involved in the Carbohydrate degradation; glycolysis; D-glyceraldehyde 3-phosphate and glycerone phosphate from D-glucose: step 3/4.](http://www.grenoble.prabi.fr/obiwarehouse/unipathway?upid=UPA00109&entryac=B3WE64) | 34 |
| 25 | L-lactate dehydrogenase 1 | [*Enterococcus faecalis*](http://www.uniprot.org/taxonomy/1351) | ldh1 | Q839C1 | 35465,2383 | 4,77 | 7 | 196 | 100 | [It involved in the fermentation; pyruvate fermentation to lactate; (S)-lactate from pyruvate: step 1/1.](http://www.grenoble.prabi.fr/obiwarehouse/unipathway?upid=UPA00554&entryac=Q839C1) | 3 |
| 26 | Enolase | [*Aeromonas hydrophila* subsp. *hydrophila*](http://www.uniprot.org/taxonomy/380703) | eno | A0KGH3 | 45691,5117 | 5,24 | 12 | 71 | 97 | Catalyzes the reversible conversion of 2-phosphoglycerate into phosphoenolpyruvate. It is essential for the degradation of carbohydrates via glycolysis | 15 |
| 26 | Enolase | [*Escherichia coli* O127:H6](http://www.uniprot.org/taxonomy/574521) | eno | B7UHJ5 | 45626,4219 | 5,32 | 14 | 90 | 100 | Catalyzes the reversible conversion of 2-phosphoglycerate into phosphoenolpyruvate. It is essential for the degradation of carbohydrates via glycolysis. It is also a component of the RNA degradosome, a multi-enzyme complex involved in RNA processing and messenger RNA degradation. | 35 |
| 27 | Elongation factor Tu 1 | [*Escherichia coli* O9:H4](http://www.uniprot.org/taxonomy/331112) | tuf1 | A8A5E6 | 43256,3086 | 5,3 | 23 | 291 | 100 | This protein promotes the GTP-dependent binding of aminoacyl-tRNA to the A-site of ribosomes during protein biosynthesis | 32 |
| 27 | Elongation factor Tu | [*Salmonella choleraesuis*](http://www.uniprot.org/taxonomy/28901) | tuf1 | Q57H76 | 43256,3086 | 5,3 | 23 | 291 | 100 |  | 36 |
| 27 | Elongation factor Tu | [*Klebsiella pneumoniae* subsp. *pneumoniae*](http://www.uniprot.org/taxonomy/272620) | tufA | A6TEX7 | 43219,2617 | 5,29 | 21 | 247 | 100 |  | 33 |
| 27 | Elongation factor Tu | [*Erwinia carotovora subsp. atroseptica*](http://www.uniprot.org/taxonomy/29471) | tuf1 | Q6CZW6 | 43247,1992 | 5,24 | 16 | 168 | 100 |  | 37 |
| 28 | ATP synthase subunit alpha | [*Escherichia coli* O127:H6](http://www.uniprot.org/taxonomy/574521) | atpA | B7UMJ9 | 55187,75 | 5,8 | 12 | 108 | 100 | Produces ATP from ADP in the presence of a proton gradient across the membrane. The alpha chain is a regulatory subunit | 14 |
| 28 | ATP synthase subunit alpha | [*Klebsiella pneumoniae*](http://www.uniprot.org/taxonomy/507522) | atpA | B5XZM2 | 55148,7383 | 5,73 | 11 | 99 | 100 | Produces ATP from ADP in the presence of a proton gradient across the membrane. The alpha chain is a regulatory subunit | 38 |
| 28 | ATP synthase subunit alpha | [*Citrobacter koseri*](http://www.uniprot.org/taxonomy/290338) | atpA | A8ACN8 | 55244,7695 | 5,8 | 11 | 98 | 100 |  | 28 |
| 28 | ATP synthase subunit alpha | [*Klebsiella pneumoniae* subsp. *pneumoniae*](http://www.uniprot.org/taxonomy/272620) | atpA | A6TG38 | 55134,7188 | 5,73 | 10 | 91 | 100 |  | 33 |
| 28 | ATP synthase subunit alpha | [*Salmonella arizonae*](http://www.uniprot.org/taxonomy/41514) | atpA | A9MJR7 | 55094,6484 | 5,71 | 10 | 91 | 100 |  | 39 |
| 29 | D-alanine--D-alanine ligase | [*Prochlorococcus marinus*](http://www.uniprot.org/taxonomy/167546) | ddl | A3PEE0 | 40253,1992 | 6,36 | 13 | 89 | 100 | [It involved in the cell wall biogenesis and peptidoglycan biosynthesis.](http://www.grenoble.prabi.fr/obiwarehouse/unipathway?upid=UPA00219&entryac=A3PEE0) | 40 |
| 30 | Glyceraldehyde-3-phosphate dehydrogenase | [*Salmonella typhimurium*](http://www.uniprot.org/taxonomy/90371) | gapA | P0A1P0 | 35564,3086 | 6,33 | 11 | 145 | 100 | [It involved in the carbohydrate degradation; glycolysis; pyruvate from D-glyceraldehyde 3-phosphate: step 1/5.](http://www.grenoble.prabi.fr/obiwarehouse/unipathway?upid=UPA00109&entryac=P0A1P0) | 2 |
| 30 | Glyceraldehyde-3-phosphate dehydrogenase A | [*Escherichia fergusonii*](http://www.uniprot.org/taxonomy/585054) | gapA | B7LQ20 | 35518,3008 | 6,61 | 11 | 145 | 100 |  | 2 |
| 30 | Glyceraldehyde-3-phosphate dehydrogenase | [*Salmonella typhimurium*](http://www.uniprot.org/taxonomy/90371) | gapA | P0A1P0 | 35564,3086 | 6,33 | 11 | 168 | 100 |  | 2 |
| 30 | Glyceraldehyde-3-phosphate dehydrogenase A | [*Escherichia fergusonii*](http://www.uniprot.org/taxonomy/585054) | gapA | B7LQ20 | 35518,3008 | 6,61 | 11 | 168 | 100 |  | 2 |
| 30 | Glyceraldehyde-3-phosphate dehydrogenase | *Citrobacter freundii* | gap | P24748 | 31458,1504 | 5,81 | 8 | 118 | 100 |  | 2 |
| 31 | Outer membrane protein A | [*Escherichia coli*](http://www.uniprot.org/taxonomy/83333) | ompA | P0A910 | 37177,6602 | 5,99 | 9 | 110 | 100 | Required for the action of colicins K and L and for the stabilization of mating aggregates in conjugation. Serves as a receptor for a number of T-even like phages. | 12 |
| 31 | Outer membrane protein A | [*Escherichia fergusonii*](http://www.uniprot.org/taxonomy/585054) | ompA | B7LNW7 | 37678,8906 | 5,46 | 7 | 93 | 100 | Required for the action of colicins K and L and for the stabilization of mating aggregates in conjugation. Serves as a receptor for a number of T-even like phages. Also acts as a porin with low permeability that allows slow penetration of small solutes | 2 |
| 31 | Outer membrane protein A | [*Escherichia fergusonii*](http://www.uniprot.org/taxonomy/564) | ompA | P0C8Z2 | 26128,2695 | 5,14 | 6 | 93 | 100 |  | 2 |
| 31 | Citrate lyase subunit beta | [*Escherichia coli*](http://www.uniprot.org/taxonomy/83333) | citE | P0A9I1 | 33089,25 | 5,54 | 8 | 74 | 99 | Represents a citryl-ACP lyase | 26 |
| 31 | Outer membrane protein A | [*Escherichia coli*](http://www.uniprot.org/taxonomy/83333) | ompA | P0A910 | 37177,6602 | 5,99 | 12 | 132 | 100 | Required for the action of colicins K and L and for the stabilization of mating aggregates in conjugation. Serves as a receptor for a number of T-even like phages. | 12 |
| 31 | Outer membrane protein A | [*Escherichia fergusonii*](http://www.uniprot.org/taxonomy/564) | ompA | P0C8Z2 | 26128,2695 | 5,14 | 8 | 104 | 100 | Required for the action of colicins K and L and for the stabilization of mating aggregates in conjugation. Serves as a receptor for a number of T-even like phages. Also acts as a porin with low permeability that allows slow penetration of small solutes | 2 |
| 31 | Outer membrane protein A | *Citrobacter freundii* | ompA | P24016 | 25647,9609 | 4,94 | 9 | 92 | 100 |  | 2 |

**References in the additional file tables**

1. Nguyen TN, Samuelson P, Sterky F, Merle-Poitte C, Robert A, Baussant T, Haeuw JF, Uhlen M, Binz H, Stahl S: **Chromosomal sequencing using a PCR-based biotin-capture method allowed isolation of the complete gene for the outer membrane protein A of *Klebsiella pneumonia*.** *Gene* 1998, **210**:93-101.

2. Lawrence JG, Ochman H, Hartl DL**: Molecular and evolutionary relationships among enteric bacteria.** *Journal of General Microbiology* 1991, **137**:1911-1921.

3. Paulsen IT, Banerjei L, Myers GSA, Nelson KE, Seshadri R, Read TD, Fouts DE, Eisen JA, Gill SR, Heidelberg JF, Tettelin H, Dodson RJ, Umayam LA., Brinkac LM, Beanan MJ, Daugherty SC, DeBoy RT, Durkin SA, Fraser CM**: Role of mobile DNA in the evolution of vancomycin-resistant *Enterococcus faecalis*.** *Science* 2003, **299**:2071-2074.

4. Takami H, Takaki Y, Uchiyama I: **Genome sequence of *Oceanobacillus iheyensis* isolated from the Iheya Ridge and its unexpected adaptive capabilities to extreme environments.** *Nucleic Acids Res*e*arch* 2002, **30**:3927-3935.

5. Chen XH, Koumoutsi A, Scholz R, Eisenreich A, Schneider K, Heinemeyer I, Morgenstern B, Voss B, Hess WR, Reva O, Junge H, Voigt B, Jungblut PR, Vater J, Suessmuth R, Liesegang H, Strittmatter A, Gottschalk G, Borriss R: **Comparative analysis of the complete genome sequence of the plant growth-promoting bacterium *Bacillus amyloliquefaciens* FZB42.** *Nature Biotechnology* 2007, **25**:1007-1014.

6. Baba T, Kuwahara-Arai K, Uchiyama I, Takeuchi F, Ito T, Hiramatsu KJ: **Complete genome sequence of *Macrococcus caseolyticus* strain JCSCS5402, reflecting the ancestral genome of the human-pathogenic staphylococci.** *The Journal of Bacteriology* 2009, 191:1180-1190.

7. Dutka-Malen S, Molinas C, Arthur M, Courvalin P: **The VANA glycopeptide resistance protein is related to D-alanyl-D-alanine ligase cell wall biosynthesis enzymes.** *Molecular and General Genetics* 1990, **224**:364-372.

8. Beres SB, Sylva GL, Barbian KD, Lei B, Hoff JS, Mammarella ND, Liu M-Y, Smoot JC, Porcella SF, Parkins LD, Campbell DS, Smith TM, McCormick JK, Leung DYM, Schlievert PM, Musser JM: **Genome sequence of a serotype M3 strain of group A *Streptococcus*: phage-encoded toxins, the high-virulence phenotype, and clone emergence.** *Proceedings of the National Academy of Sciences U.S.A.* 2002, **99**:10078-10083.

9. Nakagawa I, Kurokawa K, Yamashita A, Nakata M, Tomiyasu Y, Okahashi N, Kawabata S, Yamazaki K, Shiba T, Yasunaga T, Hayashi H, Hattori M, Hamada S: **Genome sequence of an M3 strain of *Streptococcus pyogenes* reveals a large-scale genomic rearrangement in invasive strains and new insights into phage evolution.** *Genome Research* 2003, **13**:1042-1055.

10. Stenberg F, Chovanec P, Maslen SL, Robinson CV, Ilag L, von Heijne G, Daley DOJ: **Protein complexes of the *Escherichia coli* cell envelope.** The Journal of *Biological Chemistry* 2005, **280**:34409-34419.

11. Arora A, Abildgaard F, Bushweller JH, Tamm LK: **Structure of outer membrane protein A transmembrane domain by NMR spectroscopy.** *Nature Structural & Molecular Biology* 2001, **8**:334-338.

12. Braun G, Cole ST: **The nucleotide sequence coding for major outer membrane protein OmpA of *Shigella dysenteriae.*** *Nucleic Acids Research* 1982, **10**:2367-2378.

13. Bolotin A, Wincker P, Mauger S, Jaillon O, Malarme K, Weissenbach J, Ehrlich SD, Sorokin A: **The complete genome sequence of the lactic acid bacterium *Lactococcus lactis* ssp. lactis IL1403.** *Genome Research* 2001, **11**:731-753.

14. Iguchi A, Thomson NR, Ogura Y, Saunders D, Ooka T, Henderson IR, Harris D, Asadulghani M, Kurokawa K, Dean P, Kenny B, Quail MA, Thurston S, Dougan G, Hayashi T, Parkhill J, Frankel G: **Complete genome sequence and comparative genome analysis of enteropathogenic *Escherichia coli* O127:H6 strain E2348/69.** *The Journal of Bacteriology* 2009, **191**:347-354.

15. Seshadri R, Joseph SW, Chopra AK, Sha J, Shaw J, Graf J, Haft DH, Wu M, Ren Q, Rosovitz MJ, Madupu R, Tallon L, Kim M, Jin S, Vuong H, Stine OC, Ali A, Horneman AJ, Heidelberg JF: **Genome sequence of Aeromonas hydrophila ATCC 7966T: jack of all trades.** *The Journal of Bacteriology* 2006, **188**:8272-8282.

16. Burling FT, Kniewel R, Buglino JA, Chadha T, Beckwith A, Lima CD: Structure of *Escherichia coli* uridine phosphorylase at 2.0 A*. Acta Crystallographica Section D* 2003, **59**:73-76.

17. Veiko VP, Chebotaev DV, Ovcharova IV, Gul'Ko LB: **Protein engineering of uridine phosphorylase from *Escherichia coli* K-12. I. Cloning and expression of uridine phosphorylase genes from *Klebsiella aerogenes* and *Salmonella typhimurium* in *E. coli.*** *Bioorganicheskaia khimiia* 1998, **24**:381-387.

18. Roberts DP, Dery PD, Yucel I, Buyer J, Holtman MA, Kobayashi DY: **Role of pfkA and general carbohydrate catabolism in seed colonization by *Enterobacter cloacae*.** *Applied and Environmental Microbiology* 1999, **65**:2513-2519.

19. Ajdic DJ, McShan WM, McLaughlin RE, Savic G, Chang J, Carson MB, Primeaux C, Tian R, Kenton S, Jia HG, Lin SP, Qian Y, Li S, Zhu H, Najar FZ, Lai H, White J, Roe BA, Ferretti JJ: **Genome sequence of Streptococcus mutans UA159, a cariogenic dental pathogen.** *Proceedings of the National Academy of Sciences U.S.A.* 2002, **99**:14434-14439.

20. Mulas L, Trappetti C, Hakenbeck R, Iannelli F, Pozzi G, Davidsen TM, Tettelin H, Oggioni M: **Pneumococcal beta glucoside metabolism investigated by whole genome comparison.** Submitted (MAR-2008) to the EMBL/GenBank/DDBJ databases.

21. Xu P, Alves JM, Kitten T, Brown A, Chen Z, Ozaki LS, Manque P, Ge X, Serrano MG, Puiu D, Hendricks S, Wang Y, Chaplin MD, Akan D, Paik S, Peterson DL, Macrina FL, Buck GAJ: **Genome of the opportunistic pathogen *Streptococcus sanguinis*.** *The Journal of Bacteriology* 2007, **189**:3166-3175.

22. Hotopp JD, Censini S, Masignani V, Covacci A, Tettelin H: **Complete genome sequence of *Streptococcus pneumoniae* strain P1031.** Submitted (DEC-2007) to the EMBL/GenBank/DDBJ databases.

23. Hosaka T, Meguro T, Yamato I, Shirakihara YJ: **Crystal structure of *Enterococcus hirae* enolase at 2.8 A resolution.** *Biochemistry* 2003, 133:817-823.

24. Rusniok C: **Complete genome sequence of Listeria monocytogenes serotype 4b strain Clip81459**. Submitted (OCT-2008) to the EMBL/GenBank/DDBJ databases.

25. Zhang J, Sprung R, Pei J, Tan X, Kim S, Zhu H, Liu CF, Grishin NV, Zhao Y: Lysine acetylation is a highly abundant and evolutionarily conserved modification in *Escherichia coli*. *Molecular & Cellular Proteomics* 2009, **8**:215-225.

26. Hayashi K, Morooka N, Yamamoto Y, Fujita K, Isono K, Choi S, Ohtsubo E, Baba T, Wanner BL, Mori H, Horiuchi T: **Highly accurate genome sequences of *Escherichia coli* K-12 strains MG1655 and W3110.** *Molecular Systems Biology* 2006, **2**:E1-E5.

27. Hayashi T, Makino K, Ohnishi M, Kurokawa K, Ishii K, Yokoyama K, Han C-G, Ohtsubo E, Nakayama K, Murata T, Tanaka M, Tobe T, Iida T, Takami H, Honda T, Sasakawa C, Ogasawara N, Yasunaga T, Shinagawa H: **Complete genome sequence of enterohemorrhagic *Escherichia coli* O157:H7 and genomic comparison with a laboratory strain K-12.** *DNA Research* 2001, **8**:11-22.

28. McClelland M, Sanderson EK, Porwollik S, Spieth J, Clifton WS, Latreille P, Courtney L, Wang C, Pepin K, Bhonagiri V, Nash W, Johnson M, Thiruvilangam P, Wilson R: Submitted (AUG-2007) to the EMBL/GenBank/DDBJ databases.

29. McClelland M, Sanderson EK, Porwollik S, Spieth J, Clifton WS, Fulton B, Wollam A, Shah N, Pepin K, Bhonagiri V, Nash W, Johnson M, Thiruvilangam P, Wilson R: Submitted (JUL-2007) to the EMBL/GenBank/DDBJ databases.

30. Chiu C-H, Tang P, Chu C, Hu S, Bao Q, Yu J, Chou Y-Y, Wang H-S., Lee Y-S: **The genome sequence of *Salmonella* enterica serovar Choleraesuis, a highly invasive and resistant zoonotic pathogen.** *Nucleic Acids Research* 2005, **33**:1690-1698.

31. Teng L-J, Hsueh PR, Tsai JC, Chen P-W, Hsu J-C, Lai HC, Lee CN, Ho SW: **groESL sequence determination, phylogenetic analysis, and species differentiation for viridans group streptococci.** *Journal of Clinical Microbiology* 2002, **40**:3172-3178.

32. Rasko DA, Rosovitz MJ, Myers GSA, Mongodin EF, Fricke WF, Gajer P, Crabtree J, Sebaihia M, Thomson NR, Chaudhuri R, Henderson IR, Sperandio V, Ravel J: **The pangenome structure of *Escherichia coli*: comparative genomic analysis of *E. coli* commensal and pathogenic isolates.** *The Journal of Bacteriology* 2008, **190**:6881-6893.

33. McClelland M, Sanderson EK, Spieth J, Clifton WS, Latreille P, Sabo A, Pepin K, Bhonagiri V, Porwollik S, Ali J, Wilson RK: Submitted (SEP-2006) to the EMBL/GenBank/DDBJ databases.

34. Maze A, Boel G, Bourand A, Loux V, Gibrat JF, Zuniga M, Hartke A, Deutscher J: ***Lactobacillus casei* BL23 complete genome sequence.** Submitted (JUN-2008) to the EMBL/GenBank/DDBJ databases.

35. Iguchi A, Thomson NR, Ogura Y, Saunders D, Ooka T, Henderson IR, Harris D, Asadulghani M, Kurokawa K, Dean P, Kenny B, Quail MA, Thurston S, Dougan G, Hayashi T, Parkhill J, Frankel G: **Complete genome sequence and comparative genome analysis of enteropathogenic *Escherichia coli* O127:H6 strain E2348/69.** *The Journal of Bacteriology* 2009, **191**:347-354.

36. Chiu C-H, Tang P, Chu C, Hu S, Bao Q, Yu J, Chou Y-Y, Wang H-S, Lee Y-S: **The genome sequence of *Salmonella* enterica serovar Choleraesuis, a highly invasive and resistant zoonotic pathogen.** *Nucleic Acids Research* 2005, **33**:1690-1698.

37. Bell KS, Sebaihia M, Pritchard L, Holden MTG, Hyman LJ, Holeva MC, Thomson NR, Bentley SD, Churcher LJC, Mungall K, Atkin R, Bason N, Brooks K, Chillingworth T, Clark K, Doggett J, Fraser A, Hance Z, Toth IK: **Genome sequence of the enterobacterial phytopathogen *Erwinia carotovora* subsp. *atroseptica* and characterization of virulence factors.** *Proceedings of the National Academy of Sciences U.S.A.* 2004, **101**:11105-11110.

38. Fouts DE, Tyler HL, DeBoy RT, Daugherty S, Ren Q, Badger JH, Durkin AS, Huot H, Shrivastava S, Kothari S, Dodson RJ, Mohamoud Y, Khouri H, Roesch LFW, Krogfelt KA, Struve C, Triplett EW, Methe BA: **Complete genome sequence of the N2-fixing broad host range endophyte *Klebsiella pneumoniae* 342 and virulence predictions verified in mice.** *PLoS Genetics* 2008, 4:E1000141-E1000141.

39. Makarova KS, Slesarev A, Wolf YI, Sorokin A, Mirkin B, Koonin EV, Pavlov A, Pavlova N, Karamychev V, Polouchine N, Shakhova V, Grigoriev I, Lou Y, Rohksar D, Lucas S, Huang K, Goodstein DM, Hawkins T, Mills DA: **Comparative genomics of the lactic acid bacteria.** *Proceedings of the National Academy of Sciences U.S.A.* 2006, **103**:15611-15616.

40. Copeland A, Lucas S, Lapidus A, Barry K, Detter JC, Glavina del Rio T, Hammon N, Israni S, Dalin E, Tice H, Pitluck S, Chain P, Malfatti S, Shin M, Vergez L, Schmutz J, Larimer F, Land M, Richardson P: **Complete sequence of *Rhodopseudomonas palustris* BisB18.** Submitted (MAR-2006) to the EMBL/GenBank/DDBJ databases.

41. Bellgard MI, Wanchanthuek P, La T, Ryan K, Moolhuijzen P, Albertyn Z, Shaban B, Motro Y, Dunn DS, Schibeci D, Hunter A, Barrero R, Phillips ND, Hampson DJ: **Genome sequence of the pathogenic intestinal spirochete *Brachyspira hyodysenteriae* reveals adaptations to its lifestyle in the porcine large intestine.** *PLoS ONE* 2009, **4**:E4641-E4641.

42. Reynolds PE, Depardieu F, Dutka-Malen S, Arthur M, Courvalin P: Glycopeptide resistance mediated by enterococcal transposon Tn*1546* requires production of VanX for hydrolysis of D-alanyl-D-alanine. *Molecular Microbiology* 1994, **13**:1065-1070.

43. Wu Z, Wright GD, Walsh CT: **Overexpression, purification, and characterization of VanX, a D-, D-dipeptidase which is essential for vancomycin resistance in *Enterococcus faecium* BM4147.** *Biochemistry* 1995, 34:2455-2463.

44. Lee KB, Backer PD, Aono T, Liu CT, Suzuki S, Suzuki T, Kaneko T, Yamada M, Tabata S, Kupfer DM, Najar FZ, Wiley GB, Roe B, Binnewies T, Ussery D, Vereecke D, Gevers D, Holsters M, Oyaizu H: **Complete genome sequence of the nitrogen-fixing bacterium *Azorhizobium caulinodans* ORS571**. Submitted (APR-2007) to the EMBL/GenBank/DDBJ databases.

45. Shibata C, Ehara T, Tomura K, Igarashi K, Kobayashi H: Gene structure of *Enterococcus hirae* (*Streptococcus faecalis*) F1F0-ATPase, which functions as a regulator of cytoplasmic pH. *The Journal of Bacteriology* 1992, **174**:6117-6124.

46. Sumby P, Porcella SF, Madrigal AG, Barbian KD, Virtaneva K, Ricklefs SM, Sturdevant DE, Graham MR, Vuopio-Varkila J, Hoe NP, Musser JM: **Evolutionary origin and emergence of a highly successful clone of serotype M1 group A Streptococcus involved multiple horizontal gene transfer events.** *The Journal of Infectious Disease* 2005, **192**:771-782.

47. Quivey RG Jr, Faustoferri RC, Belli WA, Flores JS: **Polymerase chain reaction amplification, cloning, sequence determination and homologies of streptococcal ATPase-encoding DNAs.** *Gene* 1991, **97**:63-68.

48. Chen C, Tang J, Dong W, Wang C, Feng Y, Wang J, Zheng F, Pan X, Liu D, Li M, Song Y, Zhu X, Sun H, Feng T, Guo Z, Ju A, Ge J, Dong Y, Yu J: **A glimpse of streptococcal toxic shock syndrome from comparative genomics of *S. suis* 2 Chinese isolates.** *PLoS ONE* 2007, **2**:E315-E315.

49. Takeuchi F, Watanabe S, Baba T, Yuzawa H, Ito T, Morimoto Y, Kuroda M, Cui L, Takahashi M, Ankai A, Baba S, Fukui S, Lee JC, Hiramatsu K: **Whole-genome sequencing of *Staphylococcus haemolyticus* uncovers the extreme plasticity of its genome and the evolution of human-colonizing staphylococcal species.** *The Journal of Bacteriology* 2005, **187**:7292-7308.

50. Kettler GC, Martiny AC, Huang K, Zucker J, Coleman ML, Rodrigue S, Chen F, Lapidus A, Ferriera S, Johnson J, Steglich C, Church GM, Richardson P, Chisholm SW: **Patterns and implications of gene gain and loss in the evolution of *Prochlorococcus*.** *PLoS Genetics* 2007, **3**:2515-2528.

51. Morita H, Toh H, Fukuda S, Horikawa H, Oshima K, Suzuki T, Murakami M, Hisamatsu S, Kato Y, Takizawa T, Fukuoka H, Yoshimura T, Itoh K, O'Sullivan DJ, McKay LL, Ohno H, Kikuchi J, Masaoka T, Hattori M: **Comparative genome analysis of *Lactobacillus reuteri* and *Lactobacillus fermentum* reveal a genomic island for reuterin and cobalamin production.** *DNA Research* 2008, **15**:151-161.

52. Arena ME, Manca de Nadra MC, Munoz R: **The arginine deiminase pathway in the wine lactic acid bacterium *Lactobacillus hilgardii* X1B: structural and functional study of the *arc*ABC genes.** *Gene* 2002, **301**:61-66.

53. Copeland A, Lucas S, Lapidus A, Barry K, Detter JC, Glavina del Rio T, Hammon N, Israni S, Dalin E, Tice H, Pitluck S, Chain P, Malfatti S, Shin M, Vergez L, Schmutz J, Larimer F, Land M, Richardson P: **Complete sequence of chromosome of Nitrobacter hamburgensis X14.** Submitted (MAR-2006) to the EMBL/GenBank/DDBJ databases.

54. Duez C, Thamm I, Sapunaric F, Coyette J, Ghuysen J-M: **The division and cell wall gene cluster of *Enterococcus hirae* S185.** *DNA Sequencing* 1998, **9**:149-161.
