# Supplementary material for: Proteomic characterization of vanA-containing Enterococcus recovered from Seagulls at the Berlengas Natural Reserve, W Portugal
Source: Proteome Sci. 2010 Sep 21;8:48. doi: 10.1186/1477-5956-8-48 (PMC2954869; doi:10.1186/1477-5956-8-48)
Supplement: Additional file 4 — Identification of proteins from vanA E. faecium SG 41 isolate using2-DE gels and MALDI-TOF sequencing results. Spot identification, protein description, species which was already isolated, protein name, accession number, protein MW and PI, peptide count, protein score, information about, and references (Additional file legend). [file 1477-5956-8-48-S4.DOC]

**Additional file 4:** Protein spots identification of 2-DE gels and MALDI-TOF sequencing results from *vanA E. faecium* SG 41 isolate.

| ***Spot*** | ***Protein Description*** | ***Species*** | ***Protein Name*** | ***Accession Number*** | ***Protein MW*** | ***Protein PI*** | ***Peptide Count*** | ***Protein Score*** | ***Protein Score C.I. %*** | ***Information*** | ***References*** |
| --- | --- | --- | --- | --- | --- | --- | --- | --- | --- | --- | --- |
| 1 | Inosine-5'-monophosphate dehydrogenase | *Streptococcus pyogenes* serotypeM3 | guaB | Q8K5G1 | 52773,26172 | 5,72 | 7 | 90 | 100 | Required for the purine metabolism; XMP biosynthesis via de novo pathway; XMP from IMP: step 1/1. | 8, 9 |
| 2 | Threonyl-tRNA synthetase | [*Ehrlichia chaffeensis*](http://www.uniprot.org/taxonomy/205920) | thrS | Q2GI92 | 73237,52344 | 6,94 | 14 | 69 | 96 | It involved in the ATP and zinc ion binding and the threonine-tRNA ligase activity. | 41 |
| 3 | Uracil phosphoribosyltransferase | [*Lactococcus lactis* subsp. *cremoris*](http://www.uniprot.org/taxonomy/272622) | upp | Q02WM7 | 23232,55078 | 6 | 4 | 73 | 99 | [Pyrimidine metabolism; CTP and UMP biosynthesis via salvage pathway](http://www.grenoble.prabi.fr/obiwarehouse/unipathway?upid=UPA00579&entryac=Q02WM7) | 39 |
| 3 | Uracil phosphoribosyltransferase | *Lactococcus lactis* subsp. *lactis* | upp | Q9CEC9 | 23215,59961 | 6,54 | 5 | 82 | 100 | Involved in the pyrimidine metabolism; UMP biosynthesis via salvage pathway; UMP from uracil: step 1/1. | 13 |
| 3 | Uracil phosphoribosyltransferase | [*Lactococcus lactis* subsp. *cremoris*](http://www.uniprot.org/taxonomy/272622) | upp | Q02WM7 | 23232,55078 | 6 | 4 | 74 | 99 | [Pyrimidine metabolism; CTP and UMP biosynthesis via salvage pathway](http://www.grenoble.prabi.fr/obiwarehouse/unipathway?upid=UPA00579&entryac=Q02WM7) | 39 |
| 4 | Ribosome-recycling factor | *Enterococcus faecalis* | frr | Q831V2 | 20790,96094 | 5,21 | 6 | 84 | 100 | Responsible for the release of ribosomes from messenger RNA at the termination of protein biosynthesis. Could augment the efficiency of translation by recycling ribosomes from one round of translation to another | 3 |
| 4 | DNA-directed RNA polymerase subunit beta' | [*Rhodopseudomonas palustris*](http://www.uniprot.org/taxonomy/316056) | rpoC | Q211D9 | 155957,2344 | 8,68 | 25 | 86 | 100 | DNA-dependent RNA polymerase catalyzes the transcription of DNA into RNA using the four ribonucleoside triphosphates as substrates | 40 |
| 5 | 50S ribosomal protein L28 | [*Brachyspira hyodysenteriae*](http://www.uniprot.org/taxonomy/565034) | rpmB | C0R0R3 | 6823,660156 | 10,18 | 9 | 68 | 95 | Involved in the translation | 41 |
| 5 | D-alanyl-D-alanine dipeptidase | [*Enterococcus faecium*](http://www.uniprot.org/taxonomy/1352) | vanX | Q06241 | 23365,34961 | 5,58 | 13 | 170 | 100 | Hydrolyzes D-Ala-D-Ala. Has a 250-fold differential in catalytic efficiency for hydrolysis of D-Ala-D-Ala versus D-Ala-D-lactate. Thus D-Ala-D-lactate remains intact for subsequent incorporation into peptidoglycan precursors that terminate in the depsipeptide D-Ala-D-lactate rather than the dipeptide D-Ala-D-Ala, thereby preventing vancomycin binding. | 42, 43 |
| 6 | GTP-binding protein engA | [*Azorhizobium caulinodans*](http://www.uniprot.org/taxonomy/438753) | engA | A8HVL5 | 49272,92969 | 5,78 | 12 | 69 | 96 | Involved in the GTP binding | 44 |
| 7 | ATP synthase subunit beta | [*Enterococcus hirae*](http://www.uniprot.org/taxonomy/1354) | atpD | P43451 | 50999,26953 | 4,74 | 17 | 321 | 100 | Produces ATP from ADP in the presence of a proton gradient across the membrane. The catalytic sites are hosted primarily by the beta subunits | 45 |
| 7 | ATP synthase subunit beta | [*Streptococcus pyogenes* serotype M1](http://www.uniprot.org/taxonomy/301447) | atpD | Q9A0I7 | 51023,26953 | 4,73 | 10 | 215 | 100 |  | 46 |
| 7 | ATP synthase subunit beta | [*Enterococcus faecalis*](http://www.uniprot.org/taxonomy/1351) | atpD | Q831A5 | 50775,12109 | 4,72 | 10 | 205 | 100 |  | 3 |
| 7 | ATP synthase subunit beta | [*Streptococcus downei*](http://www.uniprot.org/taxonomy/1317) | atpD | P21933 | 8706,419922 | 4,89 | 5 | 199 | 100 |  | 47 |
| 7 | ATP synthase subunit beta | [*Streptococcus suis*](http://www.uniprot.org/taxonomy/391295) | atpD | A4VVJ9 | 50908,23047 | 4,84 | 9 | 195 | 100 |  | 48 |
| 8 | Enolase | [*Enterococcus hirae*](http://www.uniprot.org/taxonomy/1354) | eno | Q8GR70 | 46382,42969 | 4,58 | 29 | 638 | 100 | Catalyzes the reversible conversion of 2-phosphoglycerate into phosphoenolpyruvate. It is essential for the degradation of carbohydrates via glycolysis | 23 |
| 8 | Enolase | [*Enterococcus faecalis*](http://www.uniprot.org/taxonomy/1351) | eno | Q9K596 | 46482,48047 | 4,56 | 19 | 493 | 100 |  | 3 |
| 8 | Enolase | [*Streptococcus mutans*](http://www.uniprot.org/taxonomy/1309) | eno | Q8DTS9 | 46828,60938 | 4,67 | 12 | 326 | 100 |  | 19 |
| 8 | L-lactate dehydrogenase 1 | [*Enterococcus faecalis*](http://www.uniprot.org/taxonomy/1351) | ldh1 | Q839C1 | 35465,23828 | 4,77 | 6 | 174 | 100 | [It involved in the fermentation; pyruvate fermentation to lactate; (S)-lactate from pyruvate: step 1/1.](http://www.grenoble.prabi.fr/obiwarehouse/unipathway?upid=UPA00554&entryac=Q839C1) | 3 |
| 9 | 6-phosphofructokinase | [*Enterococcus faecalis*](http://www.uniprot.org/taxonomy/1351) | pfkA | Q836R3 | 34390,69922 | 5,55 | 12 | 179 | 100 | It involved in the carbohydrate degradation; glycolysis; D-glyceraldehyde 3-phosphate and glycerone phosphate from D-glucose: step 3/4. | 3 |
| 9 | 6-phosphofructokinase | [*Enterococcus faecalis*](http://www.uniprot.org/taxonomy/1351) | pfkA | Q836R3 | 34390,69922 | 5,55 | 12 | 251 | 100 |  | 3 |
| 10 | 60 kDa chaperonin | [*Enterococcus faecalis*](http://www.uniprot.org/taxonomy/1351) | groL | Q93EU6 | 57074,92969 | 4,64 | 12 | 156 | 100 | Prevents misfolding and promotes the refolding and proper assembly of unfolded polypeptides generated under stress conditions | 3 |
| 11 | Phosphoglycerate kinase | [*Staphylococcus haemolyticus*](http://www.uniprot.org/taxonomy/279808) | pgk | Q4L4K4 | 42406,21094 | 4,94 | 4 | 103 | 100 | [It involved in the carbohydrate degradation; glycolysis; pyruvate from D-glyceraldehyde 3-phosphate: step 2/5.](http://www.grenoble.prabi.fr/obiwarehouse/unipathway?upid=UPA00109&entryac=Q4L4K4) | 49 |
| 11 | Phosphoglycerate kinase | *Enterococcus faecalis* | pgk | Q833I9 | 42371,01172 | 4,9 | 5 | 74 | 99 |  | 3 |
| 12 | Elongation factor Ts | [*Enterococcus faecalis*](http://www.uniprot.org/taxonomy/1351) | tsf | Q831V0 | 32113,33008 | 4,87 | 8 | 77 | 99 | Associates with the EF-Tu.GDP complex and induces the exchange of GDP to GTP. It remains bound to the aminoacyl-tRNA.EF-Tu.GTP complex up to the GTP hydrolysis stage on the ribosome | 3 |
| 13 | Gamma-glutamyl phosphate reductase | [*Prochlorococcus marinus*](http://www.uniprot.org/taxonomy/59920) | proA | Q46LW0 | 47772,03906 | 5,86 | 13 | 77 | 99 | Catalyzes the NADPH dependent reduction of L-gamma-glutamyl 5-phosphate into L-glutamate 5-semialdehyde and phosphate. The product spontaneously undergoes cyclization to form 1-pyrroline-5-carboxylate. | 50 |
| 14 | NH(3)-dependent NAD(+) synthetase | [*Lactococcus lactis* subsp. *cremoris*](http://www.uniprot.org/taxonomy/272622) | nadE | Q02Z86 | 30144,69922 | 4,98 | 3 | 69 | 96 | [It involved in cofactor biosynthesis; NAD(+) biosynthesis; NAD(+) from deamido-NAD(+) (ammonia route): step 1/1.](http://www.grenoble.prabi.fr/obiwarehouse/unipathway?upid=UPA00253&entryac=Q02Z86) | 39 |
| 15 | 50S ribosomal protein L20 | [*Lactobacillus reuteri*](http://www.uniprot.org/taxonomy/557433) | rplT | B2G8A7 | 13506,41992 | 10,95 | 10 | 86 | 100 | Binds directly to 23S ribosomal RNA and is necessary for the in vitro assembly process of the 50S ribosomal subunit. It is not involved in the protein synthesizing functions of that subunit | 51 |
| 16 | 50S ribosomal protein L20 | [*Lactobacillus reuteri*](http://www.uniprot.org/taxonomy/557433) | rplT | B2G8A7 | 45846,03125 | 4,96 | 5 | 84 | 100 |  | 51 |
| 16 | Arginine deiminase | [*Lactobacillus hilgardii*](http://www.uniprot.org/taxonomy/1588) | arcA | Q8G999 | 47142,66016 | 5,4 | 4 | 78 | 99 | [It involved in the amino-acid degradation; L-arginine degradation via ADI pathway; carbamoyl phosphate from L-arginine: step 1/2.](http://www.grenoble.prabi.fr/obiwarehouse/unipathway?upid=UPA00254&entryac=Q8G999) | 52 |
| 16 | 50S ribosomal protein L3 | [*Nitrobacter hamburgensis*](http://www.uniprot.org/taxonomy/323097) | rplC | Q1QN30 | 25633,61914 | 10,07 | 11 | 71 | 97 | One of the primary rRNA binding proteins, it binds directly near the 3'-end of the 23S rRNA, where it nucleates assembly of the 50S subunit | 53 |
| 17 | 50S ribosomal protein L20 | [*Lactobacillus reuteri*](http://www.uniprot.org/taxonomy/557433) | rplT | B2G8A7 | 45846,03125 | 4,96 | 4 | 73 | 98 | Binds directly to 23S ribosomal RNA and is necessary for the in vitro assembly process of the 50S ribosomal subunit. It is not involved in the protein synthesizing functions of that subunit | 51 |
| 18 | 50S ribosomal protein L20 | [*Lactobacillus reuteri*](http://www.uniprot.org/taxonomy/557433) | rplT | B2G8A7 | 45846,03125 | 4,96 | 4 | 78 | 99 |  | 8 |
| 18 | Arginine deiminase | [*Lactobacillus hilgardii*](http://www.uniprot.org/taxonomy/1588) | arcA | Q8G999 | 47142,66016 | 5,4 | 4 | 77 | 99 | [It involved in the amino-acid degradation; L-arginine degradation via ADI pathway; carbamoyl phosphate from L-arginine: step 1/2.](http://www.grenoble.prabi.fr/obiwarehouse/unipathway?upid=UPA00254&entryac=Q8G999) | 52 |
| 19 | Cell division protein ftsZ | [*Enterococcus hirae*](http://www.uniprot.org/taxonomy/1354) | ftsZ | O08458 | 44241,53125 | 4,65 | 14 | 175 | 100 | This protein is essential to the cell-division process. It seems to assemble into a dynamic ring on the inner surface of the cytoplasmic membrane at the place where division will occur, and the formation of the ring is the signal for septation to begin. Binds to and hydrolyzes GTP. Involved in the synthesis of the septal peptidoglycan | 54 |
| 19 | ATP synthase subunit beta | [*Streptococcus downei*](http://www.uniprot.org/taxonomy/1317) | atpD | P21933 | 8706,419922 | 4,89 | 4 | 75 | 99 | Produces ATP from ADP in the presence of a proton gradient across the membrane. The catalytic sites are hosted primarily by the beta subunits | 47 |

**References in the additional file tables**

1. Nguyen TN, Samuelson P, Sterky F, Merle-Poitte C, Robert A, Baussant T, Haeuw JF, Uhlen M, Binz H, Stahl S: **Chromosomal sequencing using a PCR-based biotin-capture method allowed isolation of the complete gene for the outer membrane protein A of *Klebsiella pneumonia*.** *Gene* 1998, **210**:93-101.

2. Lawrence JG, Ochman H, Hartl DL**: Molecular and evolutionary relationships among enteric bacteria.** *Journal of General Microbiology* 1991, **137**:1911-1921.

3. Paulsen IT, Banerjei L, Myers GSA, Nelson KE, Seshadri R, Read TD, Fouts DE, Eisen JA, Gill SR, Heidelberg JF, Tettelin H, Dodson RJ, Umayam LA., Brinkac LM, Beanan MJ, Daugherty SC, DeBoy RT, Durkin SA, Fraser CM**: Role of mobile DNA in the evolution of vancomycin-resistant *Enterococcus faecalis*.** *Science* 2003, **299**:2071-2074.

4. Takami H, Takaki Y, Uchiyama I: **Genome sequence of *Oceanobacillus iheyensis* isolated from the Iheya Ridge and its unexpected adaptive capabilities to extreme environments.** *Nucleic Acids Res*e*arch* 2002, **30**:3927-3935.

5. Chen XH, Koumoutsi A, Scholz R, Eisenreich A, Schneider K, Heinemeyer I, Morgenstern B, Voss B, Hess WR, Reva O, Junge H, Voigt B, Jungblut PR, Vater J, Suessmuth R, Liesegang H, Strittmatter A, Gottschalk G, Borriss R: **Comparative analysis of the complete genome sequence of the plant growth-promoting bacterium *Bacillus amyloliquefaciens* FZB42.** *Nature Biotechnology* 2007, **25**:1007-1014.

6. Baba T, Kuwahara-Arai K, Uchiyama I, Takeuchi F, Ito T, Hiramatsu KJ: **Complete genome sequence of *Macrococcus caseolyticus* strain JCSCS5402, reflecting the ancestral genome of the human-pathogenic staphylococci.** *The Journal of Bacteriology* 2009, 191:1180-1190.

7. Dutka-Malen S, Molinas C, Arthur M, Courvalin P: **The VANA glycopeptide resistance protein is related to D-alanyl-D-alanine ligase cell wall biosynthesis enzymes.** *Molecular and General Genetics* 1990, **224**:364-372.

8. Beres SB, Sylva GL, Barbian KD, Lei B, Hoff JS, Mammarella ND, Liu M-Y, Smoot JC, Porcella SF, Parkins LD, Campbell DS, Smith TM, McCormick JK, Leung DYM, Schlievert PM, Musser JM: **Genome sequence of a serotype M3 strain of group A *Streptococcus*: phage-encoded toxins, the high-virulence phenotype, and clone emergence.** *Proceedings of the National Academy of Sciences U.S.A.* 2002, **99**:10078-10083.

9. Nakagawa I, Kurokawa K, Yamashita A, Nakata M, Tomiyasu Y, Okahashi N, Kawabata S, Yamazaki K, Shiba T, Yasunaga T, Hayashi H, Hattori M, Hamada S: **Genome sequence of an M3 strain of *Streptococcus pyogenes* reveals a large-scale genomic rearrangement in invasive strains and new insights into phage evolution.** *Genome Research* 2003, **13**:1042-1055.

10. Stenberg F, Chovanec P, Maslen SL, Robinson CV, Ilag L, von Heijne G, Daley DOJ: **Protein complexes of the *Escherichia coli* cell envelope.** The Journal of *Biological Chemistry* 2005, **280**:34409-34419.

11. Arora A, Abildgaard F, Bushweller JH, Tamm LK: **Structure of outer membrane protein A transmembrane domain by NMR spectroscopy.** *Nature Structural & Molecular Biology* 2001, **8**:334-338.

12. Braun G, Cole ST: **The nucleotide sequence coding for major outer membrane protein OmpA of *Shigella dysenteriae.*** *Nucleic Acids Research* 1982, **10**:2367-2378.

13. Bolotin A, Wincker P, Mauger S, Jaillon O, Malarme K, Weissenbach J, Ehrlich SD, Sorokin A: **The complete genome sequence of the lactic acid bacterium *Lactococcus lactis* ssp. lactis IL1403.** *Genome Research* 2001, **11**:731-753.

14. Iguchi A, Thomson NR, Ogura Y, Saunders D, Ooka T, Henderson IR, Harris D, Asadulghani M, Kurokawa K, Dean P, Kenny B, Quail MA, Thurston S, Dougan G, Hayashi T, Parkhill J, Frankel G: **Complete genome sequence and comparative genome analysis of enteropathogenic *Escherichia coli* O127:H6 strain E2348/69.** *The Journal of Bacteriology* 2009, **191**:347-354.

15. Seshadri R, Joseph SW, Chopra AK, Sha J, Shaw J, Graf J, Haft DH, Wu M, Ren Q, Rosovitz MJ, Madupu R, Tallon L, Kim M, Jin S, Vuong H, Stine OC, Ali A, Horneman AJ, Heidelberg JF: **Genome sequence of Aeromonas hydrophila ATCC 7966T: jack of all trades.** *The Journal of Bacteriology* 2006, **188**:8272-8282.

16. Burling FT, Kniewel R, Buglino JA, Chadha T, Beckwith A, Lima CD: Structure of *Escherichia coli* uridine phosphorylase at 2.0 A*. Acta Crystallographica Section D* 2003, **59**:73-76.

17. Veiko VP, Chebotaev DV, Ovcharova IV, Gul'Ko LB: **Protein engineering of uridine phosphorylase from *Escherichia coli* K-12. I. Cloning and expression of uridine phosphorylase genes from *Klebsiella aerogenes* and *Salmonella typhimurium* in *E. coli.*** *Bioorganicheskaia khimiia* 1998, **24**:381-387.

18. Roberts DP, Dery PD, Yucel I, Buyer J, Holtman MA, Kobayashi DY: **Role of pfkA and general carbohydrate catabolism in seed colonization by *Enterobacter cloacae*.** *Applied and Environmental Microbiology* 1999, **65**:2513-2519.

19. Ajdic DJ, McShan WM, McLaughlin RE, Savic G, Chang J, Carson MB, Primeaux C, Tian R, Kenton S, Jia HG, Lin SP, Qian Y, Li S, Zhu H, Najar FZ, Lai H, White J, Roe BA, Ferretti JJ: **Genome sequence of Streptococcus mutans UA159, a cariogenic dental pathogen.** *Proceedings of the National Academy of Sciences U.S.A.* 2002, **99**:14434-14439.

20. Mulas L, Trappetti C, Hakenbeck R, Iannelli F, Pozzi G, Davidsen TM, Tettelin H, Oggioni M: **Pneumococcal beta glucoside metabolism investigated by whole genome comparison.** Submitted (MAR-2008) to the EMBL/GenBank/DDBJ databases.

21. Xu P, Alves JM, Kitten T, Brown A, Chen Z, Ozaki LS, Manque P, Ge X, Serrano MG, Puiu D, Hendricks S, Wang Y, Chaplin MD, Akan D, Paik S, Peterson DL, Macrina FL, Buck GAJ: **Genome of the opportunistic pathogen *Streptococcus sanguinis*.** *The Journal of Bacteriology* 2007, **189**:3166-3175.

22. Hotopp JD, Censini S, Masignani V, Covacci A, Tettelin H: **Complete genome sequence of *Streptococcus pneumoniae* strain P1031.** Submitted (DEC-2007) to the EMBL/GenBank/DDBJ databases.

23. Hosaka T, Meguro T, Yamato I, Shirakihara YJ: **Crystal structure of *Enterococcus hirae* enolase at 2.8 A resolution.** *Biochemistry* 2003, 133:817-823.

24. Rusniok C: **Complete genome sequence of Listeria monocytogenes serotype 4b strain Clip81459**. Submitted (OCT-2008) to the EMBL/GenBank/DDBJ databases.

25. Zhang J, Sprung R, Pei J, Tan X, Kim S, Zhu H, Liu CF, Grishin NV, Zhao Y: Lysine acetylation is a highly abundant and evolutionarily conserved modification in *Escherichia coli*. *Molecular & Cellular Proteomics* 2009, **8**:215-225.

26. Hayashi K, Morooka N, Yamamoto Y, Fujita K, Isono K, Choi S, Ohtsubo E, Baba T, Wanner BL, Mori H, Horiuchi T: **Highly accurate genome sequences of *Escherichia coli* K-12 strains MG1655 and W3110.** *Molecular Systems Biology* 2006, **2**:E1-E5.

27. Hayashi T, Makino K, Ohnishi M, Kurokawa K, Ishii K, Yokoyama K, Han C-G, Ohtsubo E, Nakayama K, Murata T, Tanaka M, Tobe T, Iida T, Takami H, Honda T, Sasakawa C, Ogasawara N, Yasunaga T, Shinagawa H: **Complete genome sequence of enterohemorrhagic *Escherichia coli* O157:H7 and genomic comparison with a laboratory strain K-12.** *DNA Research* 2001, **8**:11-22.

28. McClelland M, Sanderson EK, Porwollik S, Spieth J, Clifton WS, Latreille P, Courtney L, Wang C, Pepin K, Bhonagiri V, Nash W, Johnson M, Thiruvilangam P, Wilson R: Submitted (AUG-2007) to the EMBL/GenBank/DDBJ databases.

29. McClelland M, Sanderson EK, Porwollik S, Spieth J, Clifton WS, Fulton B, Wollam A, Shah N, Pepin K, Bhonagiri V, Nash W, Johnson M, Thiruvilangam P, Wilson R: Submitted (JUL-2007) to the EMBL/GenBank/DDBJ databases.

30. Chiu C-H, Tang P, Chu C, Hu S, Bao Q, Yu J, Chou Y-Y, Wang H-S., Lee Y-S: **The genome sequence of *Salmonella* enterica serovar Choleraesuis, a highly invasive and resistant zoonotic pathogen.** *Nucleic Acids Research* 2005, **33**:1690-1698.

31. Teng L-J, Hsueh PR, Tsai JC, Chen P-W, Hsu J-C, Lai HC, Lee CN, Ho SW: **groESL sequence determination, phylogenetic analysis, and species differentiation for viridans group streptococci.** *Journal of Clinical Microbiology* 2002, **40**:3172-3178.

32. Rasko DA, Rosovitz MJ, Myers GSA, Mongodin EF, Fricke WF, Gajer P, Crabtree J, Sebaihia M, Thomson NR, Chaudhuri R, Henderson IR, Sperandio V, Ravel J: **The pangenome structure of *Escherichia coli*: comparative genomic analysis of *E. coli* commensal and pathogenic isolates.** *The Journal of Bacteriology* 2008, **190**:6881-6893.

33. McClelland M, Sanderson EK, Spieth J, Clifton WS, Latreille P, Sabo A, Pepin K, Bhonagiri V, Porwollik S, Ali J, Wilson RK: Submitted (SEP-2006) to the EMBL/GenBank/DDBJ databases.

34. Maze A, Boel G, Bourand A, Loux V, Gibrat JF, Zuniga M, Hartke A, Deutscher J: ***Lactobacillus casei* BL23 complete genome sequence.** Submitted (JUN-2008) to the EMBL/GenBank/DDBJ databases.

35. Iguchi A, Thomson NR, Ogura Y, Saunders D, Ooka T, Henderson IR, Harris D, Asadulghani M, Kurokawa K, Dean P, Kenny B, Quail MA, Thurston S, Dougan G, Hayashi T, Parkhill J, Frankel G: **Complete genome sequence and comparative genome analysis of enteropathogenic *Escherichia coli* O127:H6 strain E2348/69.** *The Journal of Bacteriology* 2009, **191**:347-354.

36. Chiu C-H, Tang P, Chu C, Hu S, Bao Q, Yu J, Chou Y-Y, Wang H-S, Lee Y-S: **The genome sequence of *Salmonella* enterica serovar Choleraesuis, a highly invasive and resistant zoonotic pathogen.** *Nucleic Acids Research* 2005, **33**:1690-1698.

37. Bell KS, Sebaihia M, Pritchard L, Holden MTG, Hyman LJ, Holeva MC, Thomson NR, Bentley SD, Churcher LJC, Mungall K, Atkin R, Bason N, Brooks K, Chillingworth T, Clark K, Doggett J, Fraser A, Hance Z, Toth IK: **Genome sequence of the enterobacterial phytopathogen *Erwinia carotovora* subsp. *atroseptica* and characterization of virulence factors.** *Proceedings of the National Academy of Sciences U.S.A.* 2004, **101**:11105-11110.

38. Fouts DE, Tyler HL, DeBoy RT, Daugherty S, Ren Q, Badger JH, Durkin AS, Huot H, Shrivastava S, Kothari S, Dodson RJ, Mohamoud Y, Khouri H, Roesch LFW, Krogfelt KA, Struve C, Triplett EW, Methe BA: **Complete genome sequence of the N2-fixing broad host range endophyte *Klebsiella pneumoniae* 342 and virulence predictions verified in mice.** *PLoS Genetics* 2008, 4:E1000141-E1000141.

39. Makarova KS, Slesarev A, Wolf YI, Sorokin A, Mirkin B, Koonin EV, Pavlov A, Pavlova N, Karamychev V, Polouchine N, Shakhova V, Grigoriev I, Lou Y, Rohksar D, Lucas S, Huang K, Goodstein DM, Hawkins T, Mills DA: **Comparative genomics of the lactic acid bacteria.** *Proceedings of the National Academy of Sciences U.S.A.* 2006, **103**:15611-15616.

40. Copeland A, Lucas S, Lapidus A, Barry K, Detter JC, Glavina del Rio T, Hammon N, Israni S, Dalin E, Tice H, Pitluck S, Chain P, Malfatti S, Shin M, Vergez L, Schmutz J, Larimer F, Land M, Richardson P: **Complete sequence of *Rhodopseudomonas palustris* BisB18.** Submitted (MAR-2006) to the EMBL/GenBank/DDBJ databases.

41. Bellgard MI, Wanchanthuek P, La T, Ryan K, Moolhuijzen P, Albertyn Z, Shaban B, Motro Y, Dunn DS, Schibeci D, Hunter A, Barrero R, Phillips ND, Hampson DJ: **Genome sequence of the pathogenic intestinal spirochete *Brachyspira hyodysenteriae* reveals adaptations to its lifestyle in the porcine large intestine.** *PLoS ONE* 2009, **4**:E4641-E4641.

42. Reynolds PE, Depardieu F, Dutka-Malen S, Arthur M, Courvalin P: Glycopeptide resistance mediated by enterococcal transposon Tn*1546* requires production of VanX for hydrolysis of D-alanyl-D-alanine. *Molecular Microbiology* 1994, **13**:1065-1070.

43. Wu Z, Wright GD, Walsh CT: **Overexpression, purification, and characterization of VanX, a D-, D-dipeptidase which is essential for vancomycin resistance in *Enterococcus faecium* BM4147.** *Biochemistry* 1995, 34:2455-2463.

44. Lee KB, Backer PD, Aono T, Liu CT, Suzuki S, Suzuki T, Kaneko T, Yamada M, Tabata S, Kupfer DM, Najar FZ, Wiley GB, Roe B, Binnewies T, Ussery D, Vereecke D, Gevers D, Holsters M, Oyaizu H: **Complete genome sequence of the nitrogen-fixing bacterium *Azorhizobium caulinodans* ORS571**. Submitted (APR-2007) to the EMBL/GenBank/DDBJ databases.

45. Shibata C, Ehara T, Tomura K, Igarashi K, Kobayashi H: Gene structure of *Enterococcus hirae* (*Streptococcus faecalis*) F1F0-ATPase, which functions as a regulator of cytoplasmic pH. *The Journal of Bacteriology* 1992, **174**:6117-6124.

46. Sumby P, Porcella SF, Madrigal AG, Barbian KD, Virtaneva K, Ricklefs SM, Sturdevant DE, Graham MR, Vuopio-Varkila J, Hoe NP, Musser JM: **Evolutionary origin and emergence of a highly successful clone of serotype M1 group A Streptococcus involved multiple horizontal gene transfer events.** *The Journal of Infectious Disease* 2005, **192**:771-782.

47. Quivey RG Jr, Faustoferri RC, Belli WA, Flores JS: **Polymerase chain reaction amplification, cloning, sequence determination and homologies of streptococcal ATPase-encoding DNAs.** *Gene* 1991, **97**:63-68.

48. Chen C, Tang J, Dong W, Wang C, Feng Y, Wang J, Zheng F, Pan X, Liu D, Li M, Song Y, Zhu X, Sun H, Feng T, Guo Z, Ju A, Ge J, Dong Y, Yu J: **A glimpse of streptococcal toxic shock syndrome from comparative genomics of *S. suis* 2 Chinese isolates.** *PLoS ONE* 2007, **2**:E315-E315.

49. Takeuchi F, Watanabe S, Baba T, Yuzawa H, Ito T, Morimoto Y, Kuroda M, Cui L, Takahashi M, Ankai A, Baba S, Fukui S, Lee JC, Hiramatsu K: **Whole-genome sequencing of *Staphylococcus haemolyticus* uncovers the extreme plasticity of its genome and the evolution of human-colonizing staphylococcal species.** *The Journal of Bacteriology* 2005, **187**:7292-7308.

50. Kettler GC, Martiny AC, Huang K, Zucker J, Coleman ML, Rodrigue S, Chen F, Lapidus A, Ferriera S, Johnson J, Steglich C, Church GM, Richardson P, Chisholm SW: **Patterns and implications of gene gain and loss in the evolution of *Prochlorococcus*.** *PLoS Genetics* 2007, **3**:2515-2528.

51. Morita H, Toh H, Fukuda S, Horikawa H, Oshima K, Suzuki T, Murakami M, Hisamatsu S, Kato Y, Takizawa T, Fukuoka H, Yoshimura T, Itoh K, O'Sullivan DJ, McKay LL, Ohno H, Kikuchi J, Masaoka T, Hattori M: **Comparative genome analysis of *Lactobacillus reuteri* and *Lactobacillus fermentum* reveal a genomic island for reuterin and cobalamin production.** *DNA Research* 2008, **15**:151-161.

52. Arena ME, Manca de Nadra MC, Munoz R: **The arginine deiminase pathway in the wine lactic acid bacterium *Lactobacillus hilgardii* X1B: structural and functional study of the *arc*ABC genes.** *Gene* 2002, **301**:61-66.

53. Copeland A, Lucas S, Lapidus A, Barry K, Detter JC, Glavina del Rio T, Hammon N, Israni S, Dalin E, Tice H, Pitluck S, Chain P, Malfatti S, Shin M, Vergez L, Schmutz J, Larimer F, Land M, Richardson P: **Complete sequence of chromosome of Nitrobacter hamburgensis X14.** Submitted (MAR-2006) to the EMBL/GenBank/DDBJ databases.

54. Duez C, Thamm I, Sapunaric F, Coyette J, Ghuysen J-M: **The division and cell wall gene cluster of *Enterococcus hirae* S185.** *DNA Sequencing* 1998, **9**:149-161.
